# Enterocyte-specific ATGL overexpression affects intestinal and systemic cholesterol homeostasis^☆^

**DOI:** 10.1016/j.bbalip.2022.159121

**Published:** 2022-02-09

**Authors:** Melanie Korbelius, Nemanja Vujić, Katharina B. Kuentzel, Sascha Obrowsky, Silvia Rainer, Guenter Haemmerle, Thomas Rülicke, Dagmar Kratky

**Affiliations:** a Gottfried Schatz Research Center, Molecular Biology and Biochemistry, Medical University of Graz, Graz, Austria; b Institute of Molecular Biosciences, University of Graz, Graz, Austria; c BioTechMed-Graz, Graz, Austria; d Department of Biomedical Sciences, University of Veterinary Medicine Vienna, Vienna, Austria

**Keywords:** ATGL, Intestine, Triglycerides, Cholesterol absorption, HDL, LXR, PPARα

## Abstract

Enterocytes of the small intestine (SI) play an important role in maintaining systemic lipid levels by regulating dietary lipid absorption and postprandial lipoprotein secretion. An excessive amount of dietary-derived triglycerides (TGs) taken up by the apical side of enterocytes or basolaterally internalized lipoprotein remnants can be transiently stored in cytosolic lipid droplets (cLDs). As mice lacking adipose TG lipase (ATGL) in the SI display massive accumulation of cLDs but also delayed cholesterol absorption, we hypothesized that SI-specific over-expression of ATGL (Atgl iTg) might have beneficial effects on lipid homeostasis in the gut and possibly throughout the body. Here, we demonstrate that Atgl iTg mice had only modestly increased enzymatic activity despite drastically elevated *Atgl* mRNA levels (up to 120-fold) on chow diet, and was highly induced upon high-fat/high-cholesterol diet (HF/HCD) feeding. Atgl iTg mice showed markedly reduced intestinal TG concentrations after acute and chronic lipid challenge without affecting chylomicron TG secretion. Circulating plasma cholesterol levels were significantly lower in Atgl iTg mice under different feeding conditions, contrasting the accelerated uptake of dietary cholesterol into the circulation after HF/HCD feeding. In the fasted state, gene expression analysis revealed modulation of PPARα and liver X receptor (LXR) target genes by an increased fatty acid release, whereas the decreased plasma cholesterol concentrations in refed mice were more likely due to changes in HDL synthesis and secretion. We conclude that ATGL, in addition to its role in TG catabolism, plays a critical role in whole-body cholesterol homeostasis by modulating PPARα and LXR signaling in intestinal enterocytes.

## 1 Introduction

The absorption of dietary fat, especially triglycerides (TGs), phospholipids (PLs), and cholesteryl esters (CEs), is a complex process in which these lipids are already metabolized in the mouth and stomach before they reach the intestinal tract. Primarily, dietary TGs are partially hydrolyzed to diglycerides (DGs) and free fatty acids (FFAs) by the action of lingual or gastric lipase in the stomach (reviewed by [1]). Both enzymes preferentially cleave short- and medium-chain TGs at the sn-3 position, yielding 1,2-DGs [2], which facilitates lipid digestion in the small intestine (SI). The majority of TG hydrolysis, however, takes place in the proximal part of the SI, the duodenum, and is mediated by pancreatic lipase. Pre-digested food from the stomach reaches the SI, causing the liberation of cholecystokinin, gallbladder emptying, the release of bile acids from the liver, and secretion of pancreatic lipase [3]. The latter, together with co-lipase, is mainly responsible for the degradation of alimentary TGs and releases FFAs and 2-monoglycerides (2-MG) (reviewed by [4]). Hydrolysis of PLs by phospholipases yields lyso-PLs and FFAs, whereas CEs are degraded to free cholesterol (FC) and FFAs by the action of cholesterol esterase. All hydrolytic products are further taken up by the absorptive enterocytes of the SI, either by protein-mediated transport involving *e.g*. cluster of differentiation 36 (CD36), several FA-binding proteins (FABPs), and the Niemann-Pick C1-like 1 (NPC1L1) protein, or by passive diffusion (reviewed by [5]). The most important step for the subsequent secretion of dietary lipids in the form of lipoproteins, which supply the peripheral organs with FFAs, is the transport of the absorbed lipids to the smooth endoplasmic reticulum. In this organelle, MGs and FC are re-esterified with FFAs by acyltransferases to TGs and CEs, respectively (reviewed by [6]). Newly synthesized lipids have three different fates:

(1) The main function of the SI is the secretion of dietary lipids in the form of lipoproteins. Therefore, both TGs and CEs are assembled with apolipoprotein B48 (ApoB48) by the microsomal transfer protein (MTP) to form chylomicrons (CM), which are secreted into the circulation *via* the lymphatic vessels and supply lipids to peripheral tissues (reviewed by [7]). Besides mediating the uptake of FA into enterocytes, the transport protein CD36 also plays an important role in CM secretion. As a consequence of FA binding to CD36, expression levels of MTP and ApoB48 rise and promote the synthesis of large CMs [8]. While CMs are the major lipoproteins produced by the SI after lipid ingestion, the intestine is also capable of synthesizing very low-density lipoprotein (VLDL)-sized particles. Formation of these particles occurs when intracellular lipid levels are too low to drive CM synthesis, such as under fasting conditions. Intestinal VLDL particles differ from CMs not only in their density and size but also in their lipid composition and protein content (reviewed by [9]). In addition to CM and VLDL particles, the SI can secrete cholesterol independently of ApoB-containing lipoproteins. Cholesterol (FC and CE) secretion within CM particles is also mediated by MTP. In addition, enterocytes can secrete high-density lipoproteins (HDL), which originally contain mainly FC (reviewed by [5]), *via* an MTP- and ApoB-independent mechanism involving ApoA1 and ATP binding cassette A1 (ABCA1), which is under tight regulation by the intestinal liver X receptor (LXR) (reviewed by [6]). Previous studies have shown that HDL produced by the SI accounts for approximately 30% of total HDL content in the circulation [10] and is able to ameliorate inflammatory responses in the liver by neutralizing gut-derived lipopolysaccharides [11].
(2) Small luminal lipid droplets (LDs) containing newly synthesized TGs and CEs remain in the lumen of the endoplasmic reticulum, which is dependent on ApoB but independent of MTP [12,13]. The role of these luminal LDs remains unclear, but it has been suggested that they may fuse with ApoB-containing lipoproteins and provide lipids for further core expansion (reviewed by [14]).
(3) Neutral lipids such as TGs and CEs accumulate mainly apically in the cytosol of enterocytes surrounded by a PL monolayer and several surface proteins, including members of the perilipin (PLIN) family. These cytosolic LDs serve as a transient lipid storage pool and are mobilized when needed (reviewed by [6]). Degradation of cytosolic LDs involves hydrolysis of TGs, transport of FFAs and MGs to the endoplasmic reticulum, and their resynthesis into TGs prior to secretion in the form of lipoproteins. Thus, lipids mobilized from cytosolic LDs contribute to the rise in plasma TG concentrations shortly after dietary lipid digestion [15,16], indicating that transient storage of excessive lipids may enhance dietary fat absorption and maintain peripheral lipid supply during fasting.

Studies on the role of adipose TG lipase (ATGL) in the degradation of intestinal LDs revealed that ATGL in mice is not involved in the mobilization of the dietary lipid storage pool [17,18], but in a lipid pool consisting of lipids derived basolaterally and reabsorbed from the circulation [18]. Of note, enterocyte-specific loss of ATGL causes severe intestinal steatosis and impaired dietary cholesterol absorption, mainly due to downregulation of intestinal peroxisome proliferator-activated receptor α (PPARα) target genes [17]. This unexpected effect on cholesterol homeostasis raised the possibility that hyperactivation of intestinal ATGL might exert beneficial effects on lipid homeostasis in the SI and in the whole body.

To specifically assess the potentially advantageous role of high levels of intestinal ATGL, we generated transgenic mice overexpressing murine ATGL exclusively in enterocytes (Atgl iTg). Our results show that the ameliorative actions of intestinal ATGL on intracellular lipid deposition are strongly dependent on the nutritional status and affect either diet-induced intestinal steatosis or intestinal and systemic cholesterol metabolism.

## 2 Materials and methods

### 2.1 Generation of transgenic mice, genotyping, and diets

To generate an intestine-specific ATGL overexpressing mouse strain, full-length mouse *Atgl* cDNA (amplified from white adipose tissue cDNA) with a C-terminal FLAG-tag was cloned into the *AgeI/XhoI* sites of the 12.4 kb villin promoter plasmid (http://www.addgene.org/19358) as previously described [19]. Transgene DNA was excised with *PmeI*, purified, and injected into pronuclei of fertilized eggs from C57BL/6NRj mice (Supplemental Fig. S1). The transgene was detected in ear-clipped genomic DNA by PCR (fwd: GACGGCCACTGCTCTCACAT; rev: CGGGAACATGGTCTCGAGTT). Five transgenic founders were identified and transgenic lines were established by backcrossing with C57BL/6 *J* mice. After analysis of *Atgl* mRNA, the line with the highest expression level was selected for further use, designated as C57BL/6 J-Tg(Atgl) 734Biat and hereinafter referred to as Atgl iTg mice.

Age- and sex-matched intestine-specific ATGL overexpressing mice (Atgl iTg) on the C57BL/6 *J* background and their WT littermates were housed in groups of 2–4 in filter-top cages (Tecniplast, Hohenpeißenberg, Germany) in a temperature-controlled environment (22 ± 1 °C; relative humidity, 45%–65%) with *ad libitum* access to food and water in a regular light-dark cycle (12 h/12 h). All experiments were performed with age-matched female mice fed standard chow (11.9% caloric intake from fat; Altromin, Lage, Germany) or a high-fat/high-cholesterol diet (HF/HCD; 34% crude fat, 1% cholesterol; Ssniff**®**, Soest, Germany) for the indicated time periods (5–12 weeks). Mice were sacrificed by cervical dislocation either after a 4 h fasting period or after refeeding (12 h fasting, 2 h refeeding). All experiments were performed in accordance with the European Directive 2010/63/EU, approved by the Division of Genetic Engineering and Animal Experiments, Austrian Federal Ministry of Education, Science and Research (Vienna, Austria; BMWFW-66.010/0154-WF/V/3b/2015).

### 2.2 Plasma lipid analyses

Plasma was isolated by submandibular blood collection and centrifugation (7 min at 5200 *xg* and 4 °C). TG, total cholesterol (TC), FC, free glycerol (FG), and non-esterified fatty acid (NEFA) concentrations were determined using enzymatic kits according to manufacturers’ protocols (DiaSys, Holzheim, Germany; Sigma-Aldrich, ST. Louis, MO; Wako Chemicals GmbH, Neuss, Germany). CE levels were calculated by subtracting FC from TC. To analyze lipid concentrations in different lipoprotein fractions, 200 μl pooled plasma were subjected to fast protein liquid chromatography (FPLC; Pharmacia P-500, Uppsala, Sweden) equipped with a Superose 6 column (Amersham Biosciences, Piscataway, NJ).

### 2.3 Lipoprotein secretion and oral lipid tolerance test

To investigate intestinal and hepatic lipoprotein secretion (CM, VLDL), 16-h fasted mice were intraperitoneally (i.p.) injected with tyloxapol (500 mg/kg body weight; Merck KGaA; Darmstadt, Germany) to inhibit peripheral lipolysis. For CM secretion, mice received an oral olive oil bolus (200 μl) 30 min after tyloxapol injection. Blood was taken prior to injection and 1, 2, and 4 h after administration. VLDL secretion was measured in plasma collected before and 2, 4, and 6 h post-injection. Plasma TG and TC concentrations were measured as described above.

### 2.4 Tissue lipid analysis

Intracellular lipid content in the intestine and liver of WT and Atgl iTg mice was determined as previously described [18].

### 2.5 Histology and Oil red O staining

All three parts of the SI (duodenum, jejunum, ileum) and livers were fixed in 4% neutral-buffered formalin (Carl Roth GmbH, Karlsruhe, Germany) for 24–48 h and stored in 30% sucrose until cryosectioning. Intestinal and hepatic sections of 7 μm were cut (HM 560 Cryo-Star; Microm International GmbH, Walldorf, Germany) and stained with Oil Red O (ORO) (Sigma-Aldrich, St. Louis, MO) and Mayer’s hematoxylin (Carl Roth) to visualize neutral lipids and nuclei, respectively. Microscopic images were taken in 20-40× magnification using an Olympus BX63 microscope (Olympus, Shinjuku, Japan) equipped with a DP73 camera.

### 2.6 BODIPY® C_12_ gavage and immunofluorescence

Mice were fasted for 16 h prior to oral administration of 1 μg BODI-PY**®** 558/568 C_12_ (#D3835, Thermo Fisher, Waltham, MA)/g body weight (BW) in 10 μl corn oil/g BW. Four hours post-gavage, mice were sacrificed and intestines and livers were used for cryosectioning. To identify cytosolic LDs, sections were rehydrated in TBS, blocked with 0.05% TBST containing 10% anti-goat serum, and stained with PLIN2 (GP46; Progen, Heidelberg, Germany) at a dilution of 1:200. Alexa Fluor 488-labeled secondary antibodies against guinea pig (PLIN2; #A-11073; Thermo Fisher, Waltham, MA) were used at a dilution of 1:250 to visualize the respective proteins.

For detection of basal ATGL expression in the SI of WT and Atgl iTg mice, cryosections were processed as described above and incubated with anti-ATGL antibody (#2138; Cell Signaling, Danvers, MA) at a dilution of 1:200. Alexa Fluor 488-labeled secondary antibody against rabbit (#A-11008; Thermo Fisher, Waltham, MA) were used at a dilution of 1:250 to visualize the respective proteins.

To stain the nuclei, sections were incubated with DAPI (Sigma-Aldrich, St.Louis, MO) at a dilution of 1:1000, mounted with antifading Dako Fluorescence Mounting Medium (Dako North America Inc., Carpinteria, CA), and analyzed using an Olympus BX63 microscope (Olympus, Shinjuku, Japan) or an Observer Z1 microscope (Zeiss, Oberkochen, Germany) equipped with an Axiocam 506 color camera (Zeiss) and a Colibri 7 light source (Zeiss).

### 2.7 Dietary Top Fluor® Cholesterol uptake

Four hour fasted mice were gavaged with 1 μg Top Fluor**®** Cholesterol (#810255P; Avanti Lipids; Birmingham, AL)/g BW in 10 μl corn oil/g BW. Mice were sacrificed 2 h post-gavage, and SI and liver samples were collected. Swiss rolls of the three intestinal parts (duodenum, jejunum, ileum) were made starting with the proximal part in the middle. Cryosections of 7 μm were cut and stained with DAPI to visualize nuclei. Micrographs were taken using an Observer Z1 microscope (Zeiss, Oberkochen, Germany) equipped with an Axiocam 506 color camera (Zeiss) and a Colibri 7 light source (Zeiss).

### 2.8 Protein quantification and western blotting

Scrapings of the intestinal mucosa were lysed by sonication (Labsonic B. Braun, Melsungen, Germany) twice for 10 s on ice. After centrifugation (3 min at 18,000 *x g*), protein concentrations were determined in the supernatant according to the method of Lowry using the DC™ Protein Assay Kit (Bio-Rad Laboratories, Hercules, CA). To prove efficient intestine-specific overexpression, 80 μg of protein were separated by SDS-PAGE and transferred onto a nitrocellulose membrane, followed by incubation with an anti-FLAG antibody (#F3165; Sigma-Aldrich, ST. Louis, MO; 1:2000) or an anti-ATGL antibody (#2138; Cell Signaling Technology; Danvers, MA; 1:200). Monoclonal anti-mouse β-actin (#A5316; Sigma-Aldrich) was used as loading control. Secondary anti-mouse (FLAG; 1:500; Dako, Glostrup, Denmark) or anti-rabbit (ATGL; 1:2500; Thermo Fisher, Waltham, MA) antibodies conjugated with HRP were visualized using the Clarity™ Western ECL Substrate Kit (Bio-Rad Laboratories) on a ChemiDoc™ MP imaging system (Bio-Rad Laboratories).

### 2.9 TG hydrolase activity assay

The assays were performed as recently described [20]. Briefly, 40 μg of protein were diluted to a final volume of 100 μl with neutral lysis buffer (containing 1 mM DTT, pH 7) and mixed with 100 μl of TG substrate [0.3 mM triolein/sample, 0.5 μCi/sample [9,10-^3^H(N)]-triolein (Perkin Elmer, Waltham, MA), 45 μM mixed micelles of phosphatidylcholine and phosphatidylinositol (3:1, w:w)] containing NEFA-free BSA at a final concentration of 2% in 100 mM phosphate buffer. After incubation for 1 h at 37 °C, the reaction was stopped by the addition of 3.25 ml stop solution (MeOH:CHCl_3_:n-heptane, 10:9:7, v:v:v) and 1 ml of 100 mM potassium carbonate (pH 10.5). Samples were vortexed for 10–15 s and centrifuged (15 min at 800 *xg* and 4 °C). Radioactivity in 1 ml of the upper phase was determined by liquid scintillation counting, and the release of FAs was calculated [21].

### 2.10 RNA isolation, reverse transcription, and quantitative real-time PCR

Tissues were lysed (Precellys; Bertin Instruments, Montigny-le-Bretonneux, France) and RNA was extracted using TRIsure™ reagent according to the manufacturer’s protocol (Meridian Bioscience, Cincinnati, OH). Two micrograms of RNA were reverse transcribed using the High Capacity cDNA Reverse Transcription Kit (Applied Biosystems, Carlsbad, CA). Quantitative real-time PCR was performed on a CFX96 Real-Time PCR detection system (Bio-Rad Laboratories, Hercules, CA) using the GoTaq**®** qPCR Mastermix (Promega, Madison, WI). Samples were analyzed in duplicate and normalized to *cyclophilin A* expression as housekeeping gene. For comparison between different cohorts, samples were normalized to *β-actin*. Expression levels and associated statistical parameters were determined using the 2^-ΔΔCT^ method. Primer sequences are listed in Supplemental Table 1.

### 2.11 Acute lipid absorption (CM secretion)

Dietary lipid absorption of TG and cholesterol was performed as previously described [22]. Briefly, mice fed chow diet or HF/HCD for 5 weeks were fasted for 4 h, i.p. injected with 500 mg tyloxapol/kg body weight (Merck KGaA; Darmstadt, Germany), and gavaged with 200 μl corn oil containing 2 μCi [9,10-^3^H(N)]-triolein (Perkin Elmer, Waltham, MA) and 0.5 μCi [1-^14^C]-cholesterol (Perkin Elmer, Waltham, MA). Blood was collected 150 and 300 min post-gavage and mice were sacrificed 5 h after administration of the oil bolus. Stomach, duodenum, jejunum, ileum, liver, and feces were collected, lyophilized for 24 h, digested in 1 ml of 1 M NaOH, and radioactivity was measured by liquid scintillation counting.

### 2.12 Incorporation of acetic acid into lipid classes

*De novo* lipid synthesis was measured as previously described [22]. Briefly, mice were fasted for 4 h and then injected i.p. with 200 μl of PBS containing 5 μCi of [^14^C]**-**sodium acetate. One hour after injection, the organs were harvested and plasma radioactivity was measured by liquid scintillation counting. Plasma pools were subjected to FPLC as described above, and radioactivity was determined in each fraction. For determination of the lipid distribution into different lipid classes, lipids were extracted from 20 mg of lyophilized tissue using CHCl_3_:MeOH (2:1, *v*/v) as described elsewhere [18]. Dried lipid extracts were dissolved in 40 μl human serum (1:1 with CHCl_3_:MeOH) and loaded on a TLC plate. Lipids were separated with n-hexane/diethylether/acetic acid (70:30:1, v/v/v), corresponding bands for PL, FC, FFA, TG, and CE were excised, and radioactivity was determined by liquid scintillation counting.

### 2.13 Fatty acid oxidation in isolated primary enterocytes

Enterocytes were isolated as previously described [23]. Briefly, the jejunal segment was washed with Buffer A (115 mM NaCl, 5.4 mM KCl, 0.96 mM NaH_2_PO_4_, 26.19 mM NaHCO_3_, 5.5 mM glucose), after which one end of the jejunum was tied and the lumen was filled with Buffer B (67.5 mM NaCl, 1.5 mM KCl, 0.96 mM NaH_2_PO_4_, 26.19 mM NaHCO_3_, 27 mM sodium citrate, 5.5 mM glucose) and incubated in 0.9% NaCl at 37 **°**C for 15 min. The luminal content was discarded, filled with Buffer C (Buffer A plus 1.5 mM EDTA and 0.5 mM DTT) and incubated in 0.9% NaCl at 37 **°**C for 10 min. Afterwards, the luminal content was collected, filtered, and centrifuged at 1500 *xg* for 5 min at room temperature. All buffers were adjusted to pH 7.4 and aerated wit 95% O_2_ and 5% CO_2_ before use.

To investigate fatty acid oxidation (FAO), cell pellets were resuspended in 1 ml DMEM containing 0.5 mM carnitine, 0.4 μCi [1-^14^C]-palmitic acid (Perkin Elmer, Waltham, MA), and 100 μM palmitic acid. The filter in the middle well of the cell culture flask was saturated with 50 μl of 1 M NaOH, the flask was sealed with a rubber stopper and incubated for 90 min at 37 ° C. After termination of the reaction by the addition of 100 μl of 70% perchloric acid, ^14^CO_2_ was trapped for 2 h at 37 ° C. The radioactivity trapped by the filter paper was counted by liquid scintillation counting. Results were normalized to protein content.

### 2.14 Indirect calorimetry

Energy intake and energy expenditure were assessed using a climate-controlled indirect calorimetry system (TSE PhenoMaster, TSE Systems, Bad Homburg, Germany). WT and Atgl iTg mice fed HF/HCD for 10 weeks were single-housed in metabolic cages on a regular light-dark cycle (12 h light, 12 h dark) with free access to food and water. Locomotor activity using infrared sensor frames as well as O2 consumption and CO_2_ production were measured every 15 min. Lipid and glucose oxidation rates were calculated as previously described (reviewed by [24]).

### 2.15 LXR agonist treatment

Mice fed chow diet *ad libitum* received 10 mg/kg BW of the LXR agonist GW3965 (G930985; Toronto Research Chemicals, Toronto, Canada) in 10 μl/kg BW corn oil supplemented with 1% cholesterol or vehicle control (corn oil +1% cholesterol) by gavage. Blood was drawn in the 4-h fasted state at baseline and after 9 days of treatment, as previously described [25].

### 2.16 Statistical analyses

Statistical analyses were performed using GraphPad Prism 5.0 software. Significance was calculated by unpaired Student’s *t*-test or ANOVA followed by Bonferroni post-hoc tests. Data are shown as mean ± SD. For statistical analysis of mRNA expression, values were calculated using the 2^-ΔΔCT^ method and represented as mean + SD. The following levels of statistical significance were used: *, *p* < 0.05; **, *p* ≤ 0.01; ***, *p* ≤ 0.001 for comparison between WT and Atgl iTg mice and §, p < 0.05; §§, p ≤ 0.01; §§§, p ≤ 0.001 for comparison between treated and untreated mice. All graphs were sorted according to a uniform color code, with black bars representing WT mice and white bars representing Atgl iTg mice.

## 3 Key resources table

**Table:** 

Reagent or resource	Source	Identifier
Antibodies		
ATGL Antibody	Cell Signaling Technology	Cat#2138 RRID:AB_2167955
Anti-FLAG Antibody	Sigma-Aldrich	Cat#F3165 RRID:AB259529
Anti-β-Actin antibody	Sigma-Aldrich	Cat#A5316 RRID:AB476743
Anti-Perilipin 2, N-terminusaa 1-16	Progen	Cat#GP46
Rabbit anti-Mouse IgG (HRP)	Dako	Cat#P0260 RRID:AB_2687969
Goat anti-Rabbit IgG (HRP)	Thermo Fisher Scientific	Cat#31460 RRID:AB_228341
Goat anti-Guinea Pig IgG, Alexa Fluor® 488	Thermo Fisher Scientific	Cat#A11073 RRID:AB_2534117
Goat anti-Rabbit IgG, Alexa Fluor® 488	Thermo Fisher Scientific	Cat#A11008 RRID:AB_143165
Critical Commercial Assays		
Triglycerides FS	DiaSys Diagnostic Systems GmbH	Cat#157609910023
Cholesterol FS	DiaSys Diagnostic Systems GmbH	Cat#113009910023
Free Cholesterol FS	DiaSys Diagnostic Systems GmbH	Cat#113609910930
Free Glycerol Reagent	Sigma-Aldrich	Cat#F6428 Cat#434-91,795,
NEFA-HR(2)	Wako Chemicals GmbH	Cat#436-91,995, Cat#270-77,000
Bio-Rad Protein Assay	Bio-Rad Laboratories	Cat#500-0112
Plasmids		
12.4kbVillin-ΔATG	Addgene	Cat#19358 RRID:Addgene_19,358
Experimental Models: Organisms/Strains		
Mouse: Atgl iTg C57BL/6 J-Tg(Atgl) 734Biat	This paper	
Oligonucleotides		
See Table S1 for list of all primer sequences used for qRT-PCR		
Chemicals		
GW3965	Toronto Research Chemicals	Cat#G930985
BODIPY® 558/568 Cļ2	Thermo Fisher Scientific	Cat#D3835
Top Fluor® Cholesterol	Avanti Lipids	Cat#810255P
Software and Algorithms		
Graph Pad Prism 5	GraphPad Software	https://www.graphpad.com
Image J	Image J	https://imagej.net
ZEN 3.1 (Blue Edition)	Carl Zeiss Microscopy	https://www.zeiss.at

## 4 Results

### 4.1 Slightly elevated enzymatic activity despite massively increased Atgl transgene expression

**Fig. 1:**
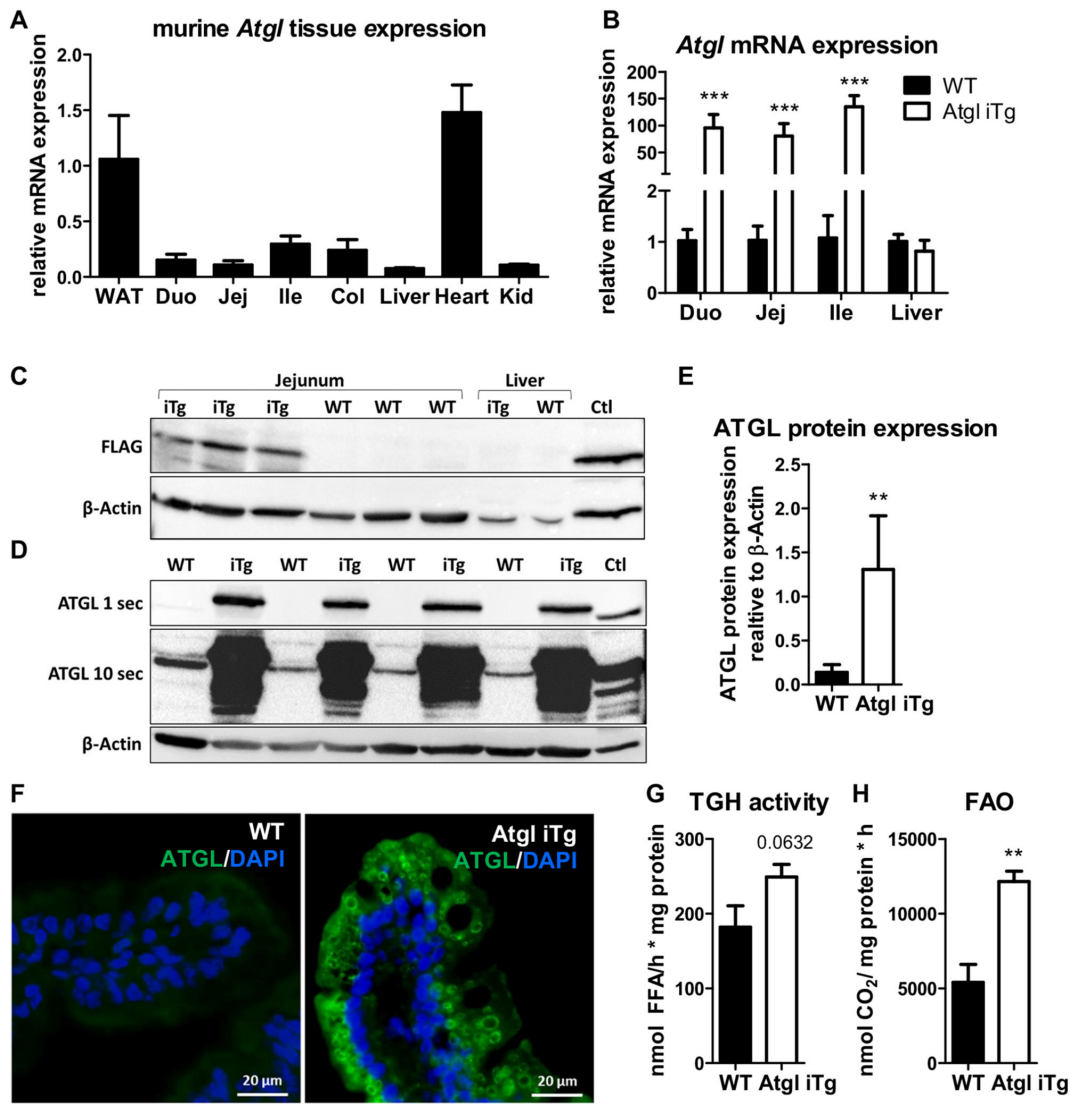
Efficient intestine-specific overexpression of ATGL. (A) *Atgl* expression in tissues of WT mice: WAT, white adipose tissue; Duo, duodenum; Jej, jejunum; Ile, ileum; Col, colon; Kid, kidney. (B) Gene expression of *Atgl* in the small intestine and liver of WT and Atgl iTg mice after 8 weeks of HF/HCD feeding, normalized to *cyclophilin A*. (C) Western blot analysis of jejunal and hepatic tissue lysates to detect FLAG-tagged ATGL. The FLAG-tagged Ces2c construct served as a positive control (Ctl) and β-actin was determined as loading control. (D) Western blot analysis and (E) corresponding quantification of isolated jejunal enterocytes to detect total ATGL with exposure times of 1 s and 10 s. Tissue lysate of WT WAT served as a positive control (Ctl) and β-actin was determined as loading control. (F) Representative images of ATGL immunofluorescence staining (green) with DAPI (blue) on jejunal cryosections. Scale bar, 20 μm. (G) Jejunal neutral TG hydrolase activity and (H) fatty acid oxidation in jejunal enterocytes of chow diet-fed Atgl iTg mice and their corresponding WT littermates. Data represent mean values of 13–15 week-old female mice (*n* = 4–6) + SD. ** *p* ≤ 0.01; *** *p* ≤ 0.001.

In contrast to white adipose tissue (WAT), *Atgl* expression is relatively low in the SI (Fig. 1A). To confirm our intestine-specific overexpression of the ATGL transgene, we analyzed mRNA expression in all three parts of the SI (duodenum, jejunum, ileum) and livers of HF/HCD-fed WT and Atgl iTg mice. *Atgl* expression was increased ~100-fold in the entire SI without effects on hepatic expression (Fig. 1B). To solely measure protein expression of the *Atgl* transgene (Supplemental Fig. S1), we performed Western blot analysis of the FLAG-tagged ATGL in jejunal and hepatic tissue lysates, resulting in the expression of a 55.5 kDa protein (ATGL 54 kDa, FLAG-Tag 1.5 kDa). FLAG expression was detected exclusively in the jejunum of transgenic mice, whereas no expression was observed in WT jejunum or liver (Fig. 1C). Total protein abundance of ATGL in isolated jejunal enterocytes indicated a low expression of ATGL in the intestines of WT mice that became apparent only after prolonged exposure, but a 9.4-fold increase in total ATGL protein abundance in Atgl iTg mice (Fig. 1D, E). Consistent with this finding, immunofluorescence staining against ATGL revealed almost undetectable fluorescence in WT mice, but circular structures within enterocytes that were positive for ATGL in Atgl iTg mice and most probably reflected cytosolic LDs (Fig. 1F). Despite the drastic upregulation of *Atgl* mRNA and efficient overexpression of ATGL protein, neutral TG hydrolase activity was increased by only 1.4-fold in the jejunum of Atgl iTg mice (Fig. 1G). This indicated that increased gene or protein expression does not reflect the enzymatic activity of ATGL, possibly due to the limited availability of its co-activator CGI-58.

However, the fatty acid oxidation (FAO) rate in isolated jejunal enterocytes was increased by 2.2-fold in Atgl iTg mice (Fig. 1H), suggesting a role for intestinal ATGL in providing FFAs for energy production.

### 4.2 Intestinal ATGL overexpression does not affect ApoB-dependent lipoprotein secretion but reduces circulating cholesterol concentrations

**Fig. 2:**
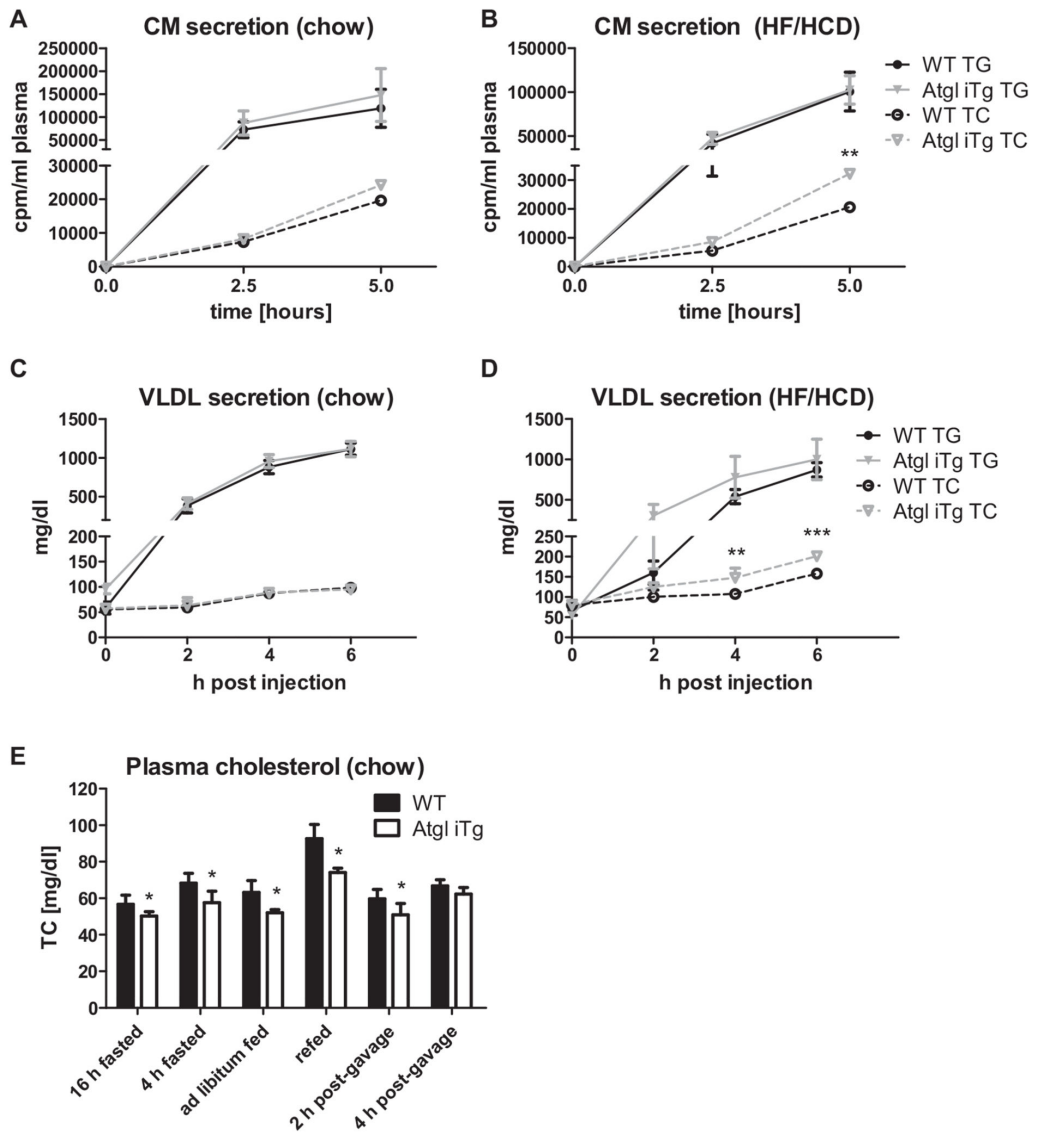
Increased CM-TC secretion in HF/HCD-fed Atgl iTg mice. Atgl iTg and control mice were fasted for 4 h prior to i.p. administration of tyloxapol. Secretion of CM-TG and CM-TC 2.5 and 5 h after an oral lipid bolus containing [^3^H]-triolein and [^14^C]-cholesterol in (A) chow diet- and (B) 5-week HF/HCD-fed WT and Atgl iTg mice. (C, D) VLDL-TG and VLDL-TC concentrations measured before, 2, 4, and 6 h after the administration of tyloxapol in (C) chow diet- and (D) 5-week HF/HCD-fed mice. (E) Plasma cholesterol levels in chow diet-fed mice under various feeding conditions. Data represent mean values of 16-21 week-old female mice (n=3-7) ± SD. * p < 0.05; ** p ≤ 0.01; *** p ≤ 0.001.

In addition to providing FFAs for energy production, ATGL could also release FFAs for re-esterification into lipoproteins or for activation of nuclear receptors like PPARs. In line with unaltered CM secretion in mice with intestinal ATGL deficiency [17,18], overexpression of ATGL also resulted in comparable CM secretion when mice were fed chow diet (Fig. 2A). Interestingly, we observed increased cholesterol release in CMs when mice were fed HF/HCD (Fig. 2B), suggesting accelerated dietary cholesterol absorption. Secretion of TG and TC within VLDL particles remained comparable between the genotypes on chow diet (Fig. 2C), but Atgl iTg mice showed elevated VLDL-cholesterol secretion after HF/HCD feeding (Fig. 2D). In addition, we found a significant decrease in circulating cholesterol in chow diet-fed Atgl iTg mice in various feeding conditions, which was mainly attributable to a reduction in CE concentrations. After overnight fasting, Atgl iTg mice displayed a 13% reduction of TC in the plasma, which was even more pronounced after 2 h of refeeding (—20%) (Fig. 2E, Table 1).

### 4.3 Reduced enterocyte TG concentrations in chow diet-fed Atgl iTg mice after an acute lipid load

**Fig. 3:**
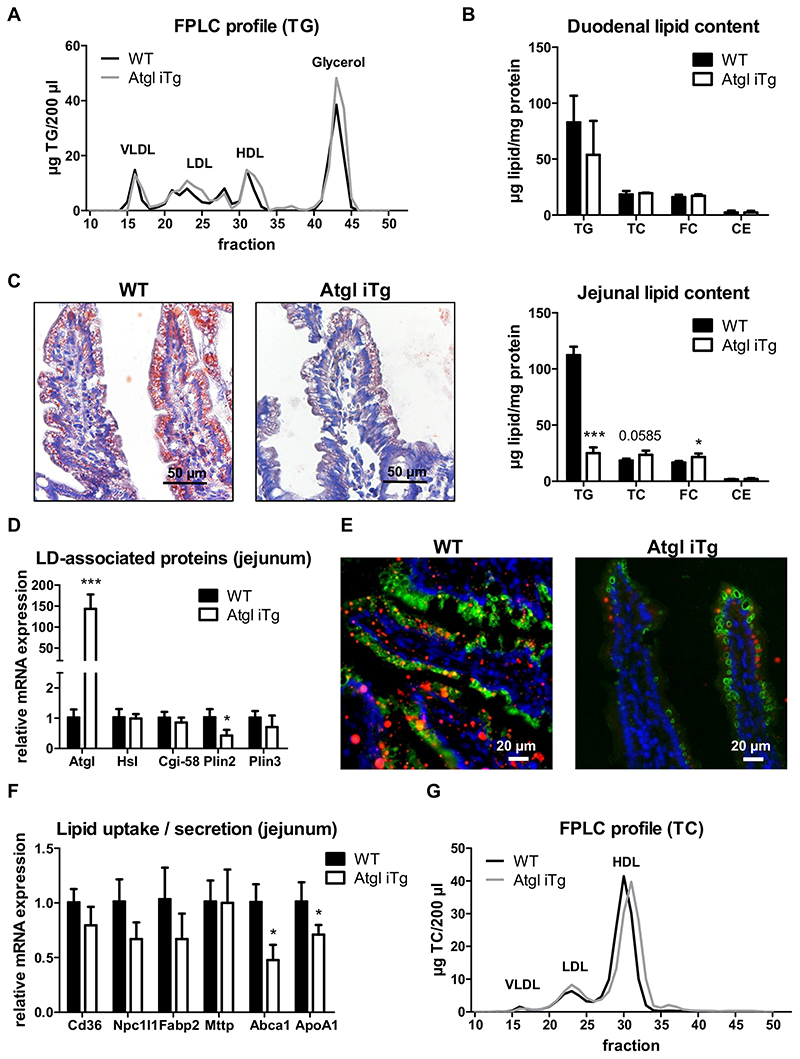
Atgl overexpression drastically reduces jejunal TG levels upon oral lipid bolus. Chow diet-fed WT and Atgl iTg mice were sacrificed 4 h after an oral oil bolus. (A) TG distribution among lipoprotein classes. (B) Duodenal lipid levels. (C) Representative oil red O staining and biochemical lipid quantification in jejuna. Scale bar, 50 μm. (D) Jejunal mRNA expression of LD-associated proteins normalized to *cyclophilin A*. (E) Jejuna of mice sacrificed 4 h post-gavage of BODIPY®-C_12_ (red) were stained with anti-PLIN2 (green) to detect LDs. Nuclei were visualized by DAPI staining (blue). Scale bar, 20 μm. (F) Jejunal mRNA expression of genes involved in cholesterol metabolism normalized to *cyclophilin A*. (G) TC distribution among lipoprotein classes. Data represent mean values of 33 week-old female mice (*n* = 3-4) + SD. * *p* < 0.05; *** *p* ≤ 0.001. (For interpretation of the references to color in this figure legend, the reader is referred to the web version of this article.)

In WAT, *Atgl* mRNA expression is induced by fasting and down-regulated upon refeeding [26]. However, intestinal *Atgl* expression is inversely regulated with the highest expression after feeding [27,28]. We therefore investigated whether even a short-term (acute) lipid challenge is sufficient to detect ameliorative effects on intestinal and systemic lipid levels in Atgl iTg mice. A previous study has shown that the size of cytosolic LDs in enterocytes increases 1.5 to 3 h post-gavage, decreases slightly from 3 to 6 h, and LDs are almost completely depleted 12 h later [29]. As we used less oil (200 μl instead of 300 μl), we decided to sacrifice the mice 4 h after the oral oil bolus to reach the peak of intestinal LDs. Atgl iTg mice displayed 30% higher circulating TG levels (Table 1), which was mainly attributable to an increase in free glycerol (Fig. 3A), indicating increased TG mobilization from intracellular LDs. While duodenal lipid concentrations remained comparable between the genotypes (Fig. 3B), jejunal lipid content as visualized by oil red O staining and lipid quantification (specifically TG concentrations) were drastically decreased by 78% in Atgl iTg mice (Fig. 3C). Downregulation of *Plin2* by 59% (Fig. 3D) indicated higher accessibility of ATGL to the LD surface [30] and potentially increased ATGL-mediated hydrolysis of cytosolic lipid stores. To confirm this observation, we stained jejunal cryosections of mice gavaged with a fluorescently-labeled FA with an anti-PLIN2 antibody. In agreement with reduced intracellular lipid deposition, we observed less abundance of fluorescent LDs, accompanied by a significant reduction in intestinal expression of PLIN2 in Atgl iTg mice (Fig. 3E). Interestingly, decreased mRNA expression of genes involved in intestinal HDL synthesis (*Abca1, ApoA1*) highlighted a possible role of intestinal ATGL in cholesterol homeostasis in the gut (Fig. 3F). Accordingly, plasma cholesterol concentrations were reduced in Atgl iTg mice (Table 1), which was attributable to a shift in the HDL cholesterol fraction indicating smaller HDL particles (Fig. 3G). However, jejunal cholesterol levels were slightly increased in Atgl iTg mice (Fig. 3B), raising the question of whether *de novo* synthesized or dietary cholesterol present in the oil gavage was responsible for this effect.

### 4.4 Accelerated dietary cholesterol absorption in Atgl iTg mice

**Fig. 4:**
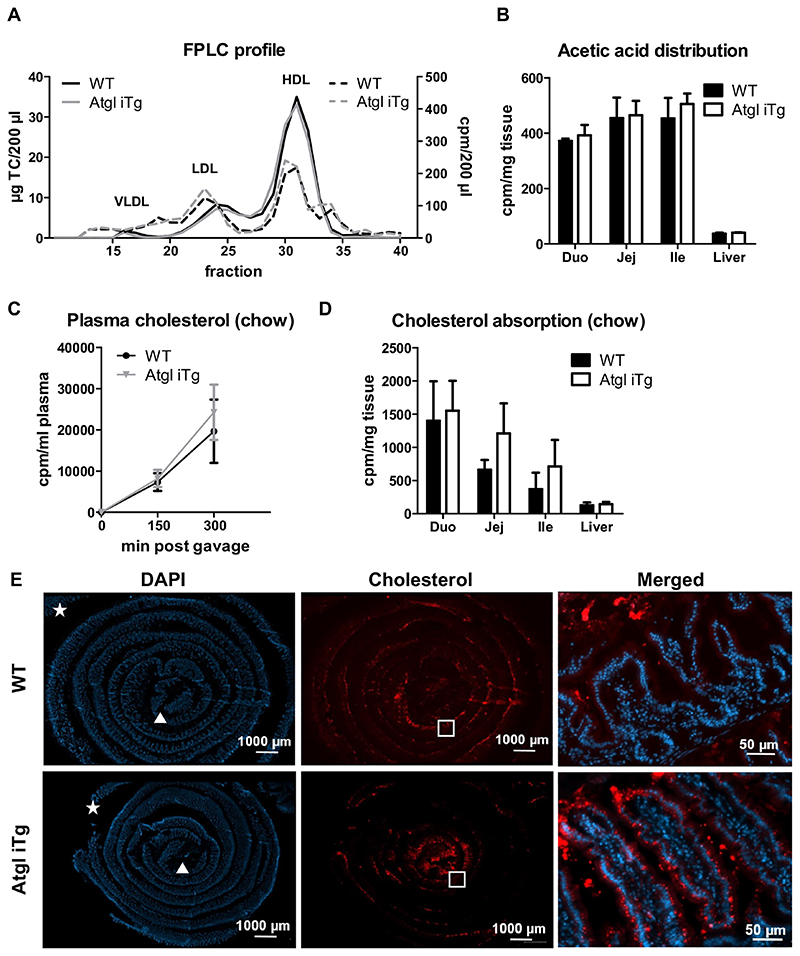
Accelerated cholesterol uptake into enterocytes of Atgl iTg mice. (A, B) Chow diet-fed mice were fasted for 4 h prior to intraperitoneal injection of [^14^C]-acetate in 200 μl PBS and sacrificed 1 h after injection. (A) Distribution of total cholesterol (solid line) and radioactivity (dashed line) in plasma lipoprotein fractions, (B) intestine, and liver. (C) Secretion of orally administered [^14^C]-cholesterol into the circulation and (D) distribution of radioactivity in intestinal and hepatic tissues. Data represent mean values of 21 week-old female mice (n = 4) ± SD. (E) Cryosections of jejunal swiss rolls (middle to outer layer: proximal to distal part) 2 h after oral administration of fluorescently-labeled cholesterol (red) with DAPI staining for nuclei (blue) in 14 week-old female mice. Triangle, proximal end; star, distal end. Scale bar, 1000 μm; magnification of square: scale bar, 50 μm. (For interpretation of the references to color in this figure legend, the reader is referred to the web version of this article.)

To study the impact of intestinal ATGL overexpression on *de novo* cholesterol synthesis, we injected mice intraperitoneally with [^14^C]-acetate. Total radioactivity in plasma (Supplemental Fig. S2A) as well as radioactivity and cholesterol content in different lipoprotein fractions (Fig. 4A) remained comparable between the genotypes. Similar incorporation of the radioactive tracer into intestinal and hepatic tissues (Fig. 4B) and unchanged distribution among different lipid classes in these tissues (Supplemental Fig. 2B-E) indicated that *de novo* synthesized cholesterol does not contribute to the observed changes in intestinal and circulating cholesterol concentrations in Atgl iTg mice. In line, mRNA expression of genes involved in cholesterol synthesis (*Hmgcr*) or esterification (*Acat2*) was unchanged in all cohorts fed chow diet (data not shown).

Another possibility for the reduced plasma cholesterol concentrations in Atgl iTg mice could be impaired dietary cholesterol uptake. Intestine-specific Atgl-deficient mice, however, showed disturbed cholesterol absorption, which was attributable to defective PPARα signaling [17]. Oral administration of [^14^C]-cholesterol resulted in comparable uptake of the radioactive tracer into the circulation (Fig. 4C) and tissues of chow-fed Atgl iTg mice; however, there was a tendency for increased deposition of cholesterol in the distal parts of the SI (Fig. 4D), which was consistent with slightly increased intestinal cholesterol levels after an acute lipid load (Fig. 3B). Visualization of fluorescently-labeled cholesterol in intestinal swiss rolls 2 h after oral administration revealed that Atgl iTg mice took up more cholesterol into the enterocytes of the distal duodenum (data not shown) and proximal jejunum compared to WT mice (Fig. 4E). Importantly, a substantial amount of cholesterol remained in the intestinal lumen, consistent with the fact that only 50% of dietary cholesterol is absorbed in the SI (reviewed by [5]).

**Fig. 5:**
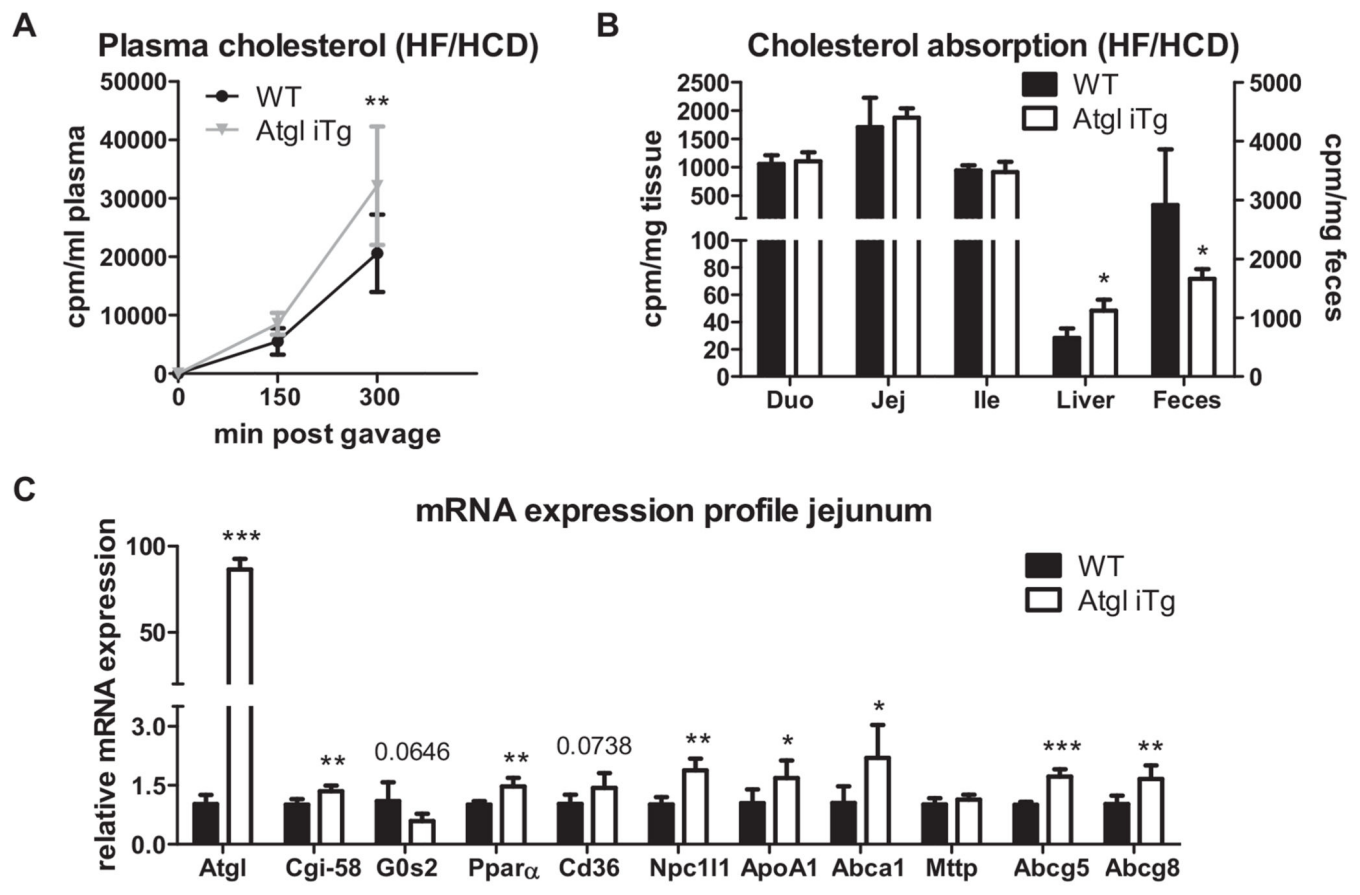
Accelerated dietary cholesterol absorption in HF/HCD-fed Atgl iTg mice. (A) Secretion of orally administered [^14^C]-cholesterol into the circulation and (B) distribution of the radioactivity in intestinal and hepatic tissues in 5-week HF/HCD-fed Atgl iTg mice. (C) Gene expression analysis of LD-associated proteins, PPARα target genes, and *Mttp* in jejuna of WT and Atgl iTg mice after 5 weeks of HF/HCD feeding, normalized to *cyclophilin A*. Data represent mean values of 12-13 week-old female mice (*n* = 4-5) ± SD. * *p* < 0.05; ** *p* ≤ 0.001; *** *p* ≤ 0.001.

Comparable to the results observed after acute lipid load, Atgl iTg mice showed accelerated cholesterol absorption from the intestinal lumen after 5 weeks of HF/HCD feeding, as indicated by a 1.6-fold increased secretion of the radioactive tracer into the bloodstream (Fig. 5A) and a 37% decrease in fecal excretion (Fig. 5B). In agreement with the lower hepatic cholesterol deposition in mice lacking ATGL [17], Atgl iTg mice had more hepatic, but unchanged intestinal cholesterol absorption (Fig. 5B). Gene expression analysis in jejuna of HF/HCD-fed Atgl iTg mice revealed a slight, but significant increase in *Cgi-58*, the co-activator of ATGL, and decreased expression of its endogenous inhibitor *G0s2*, accompanied by induction of *Ppara* and its target genes (Fig. 5C). Unchanged expression of *Mttp* in Atgl iTg mice suggested a CM-independent cholesterol release from the SI into the circulation. However, increased expression of jejunal *Npc1l1, Apoa1*, and *Abca1* (Fig. 5C) could be responsible for the observed accelerated cholesterol absorption on HF/HCD, but this observation was in contrast to the gene expression pattern we observed after an acute lipid load (Fig. 3C).

### 4.5 Intestinal ATGL overexpression improves diet-induced intestinal steatosis

**Fig. 6:**
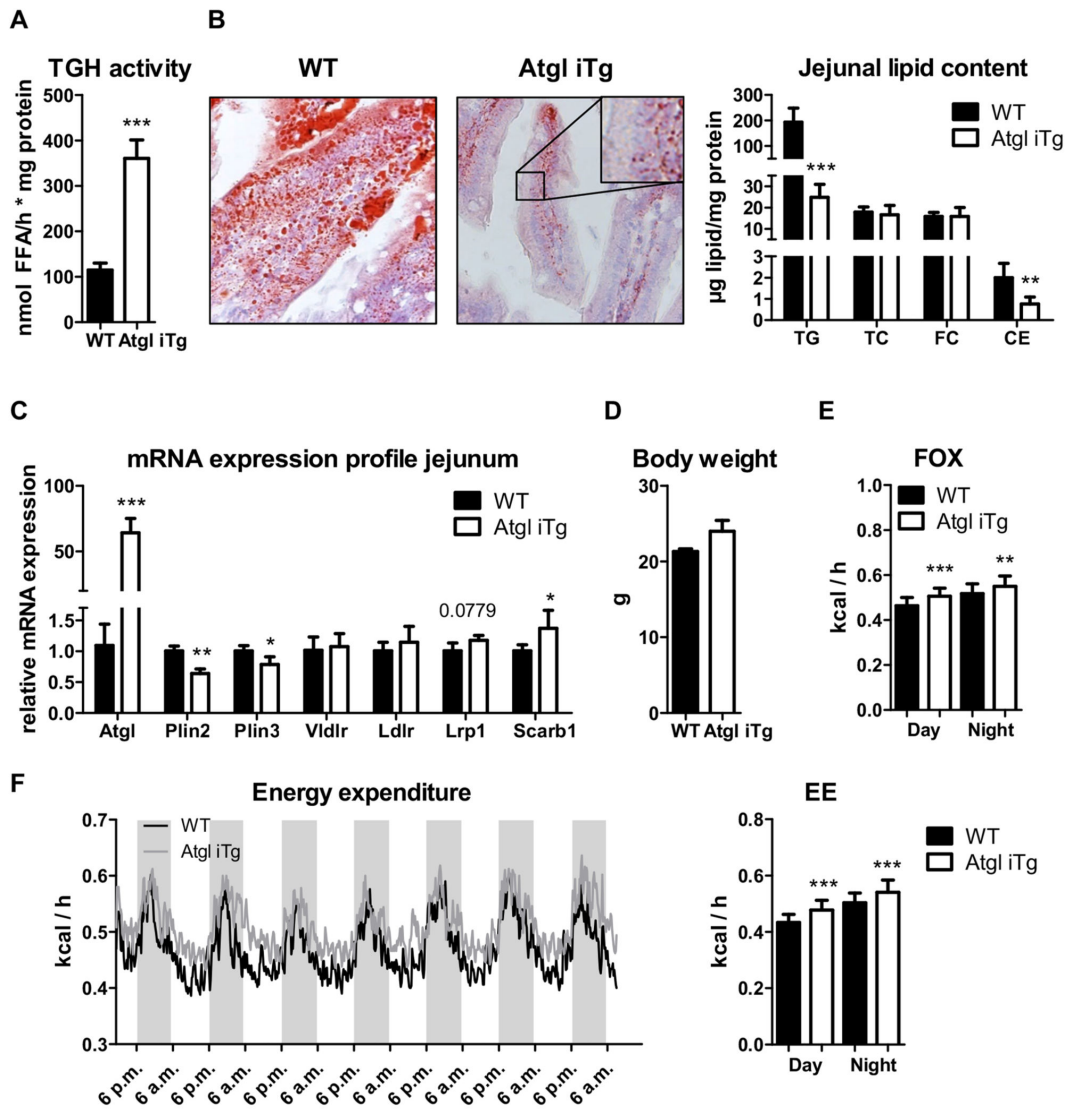
Increased energy expenditure and fatty acid utilization in HF/HCD-fed Atgl iTg mice. Jejunal (A) TG hydrolase activity and (B) lipid levels. (C) mRNA expression analysis of Plin2, Plin3, and genes involved in basolateral lipid uptake of HF/HCD-fed mice after 4 h of fasting, normalized to cyclophilin A. (D—F) After 10 weeks of HF/HCD feeding, mice were housed in metabolic cages, and fatty acid oxidation (FOX) and energy expenditure (EE) were analyzed. Data represent mean values of 16—18 week-old female mice (n = 3—5) ± SD. * p < 0.05; ** p ≤ 0.001; *** p ≤ 0.001.

Given these differences between acute and chronic lipid loading, we next studied the potential beneficial effects of ATGL overexpression under chronic HF/HCD challenge in different feeding conditions (fasted/refed). It has been previously demonstrated that intestinal *Atgl* expression is upregulated not only after refeeding, but also in mice with diet-induced obesity [28]. Slightly reduced wet and dry weight of jejunal tissue provided first evidence of beneficial effects of ATGL overexpression on intestinal steatosis after 12 weeks of HF/HCD feeding (Supplemental Fig. S3A). Body weight gain during the feeding period remained comparable between the genotypes (data not shown), but plasma TG concentrations of Atgl iTg mice were reduced by 29%, with no effect on circulating cholesterol levels after 4 h of fasting (Table 2). TG hydrolase activity in the jejunum was increased by 3.2-fold in Atgl iTg mice (Fig. 6A), suggesting higher enzymatic activity of ATGL after feeding a HF/HCD compared to chow diet (Fig. 1G). Consistent with increased TG hydrolase activity, TG content in the jejunum of Atgl iTg mice was reduced by 88%, accompanied by a 62% reduction in jejunal CE concentrations (Fig. 6B), which was already observable macroscopically (Supplemental Fig. S3B). Of note, enterocytes of WT jejuna were overloaded with lipids, whereas in Atgl iTg mice ORO-stained neutral lipids were localized exclusively on the basolateral surface of enterocytes and in the lamina propria (Fig. 6B, inset). Intracellular lipid levels in the ileum or liver remained comparable between the genotypes (data not shown). Significantly reduced expression of both *Plin2* and *Plin3* suggested facilitated hydrolysis of intestinal LDs by ATGL, further contributing to the decline in intracellular TG levels. Slightly increased jejunal mRNA expression of *Lrp1* and *Scarb1* in Atgl iTg mice indicated higher re-uptake of lipids from the bloodstream, as these lipids are the primary substrates for intestinal ATGL and are predominantly used for energy production (Fig. 6C). In agreement with these results, metabolic characterization of WT and Atgl iTg mice with comparable body weight after 10 weeks of HF/HCD feeding (Fig. 6D) showed increased fatty acid utilization, calculated from VO_2_ and VCO_2_, by 11% and 6% during the day and night, respectively (Fig. 6E); glucose oxidation and food consumption remained unchanged (data not shown). Energy expenditure was slightly increased in HF/HCD-fed Atgl iTg compared to WT mice during both light (+11%) and dark (+8%) phases (Fig. 6F). Overall locomotor activity and respiratory exchange rate (RER) remained comparable between the genotypes, however, Atgl iTg mice showed slightly higher locomotor activities on days 4 and 5 (Supplemental Fig. S3C, D).

### 4.6 HF/HCD-fed Atgl iTg mice show decreased HDL postprandially

**Fig. 7:**
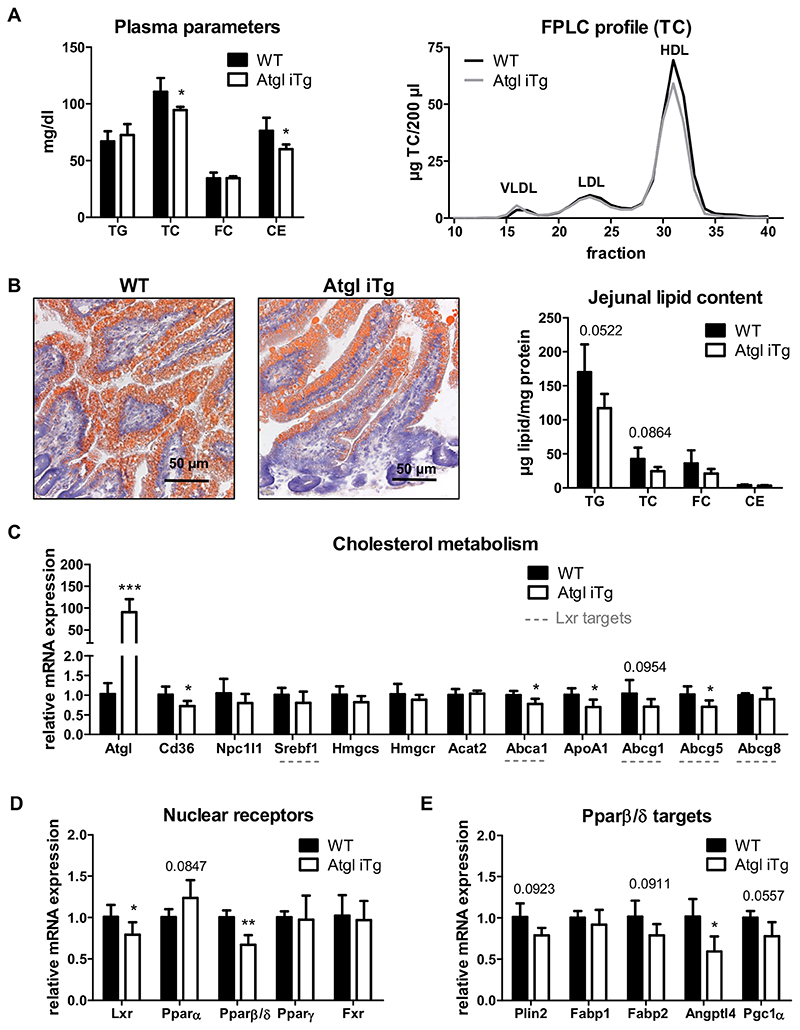
Refeeding for 2 h decreases HDL in HF/HCD-fed Atgl iTg mice. WT and Atgl iTg mice fed HF/HCD for 8 weeks were sacrificed 2 h after refeeding. (A) Plasma lipid parameters and lipoprotein profile. (B) Representative oil red O-stained sections and biochemical lipid quantification of jejuna from WT and Atgl iTg mice. Scale bar, 50 μm. Jejunal mRNA expression of (C) genes involved in intestinal cholesterol metabolism, including LXR target genes (underlined), (D) nuclear receptors, and (E) PPARβ/δ target genes, normalized to cyclophilin A as housekeeping gene. Data represent mean values of 18 week-old female mice (n = 3–5) + SD. * p < 0.05; ** p ≤ 0.01; *** p ≤ 0.001.

As intestinal ATGL expression is induced in the refed state, at least when fed chow diet [27], we next sacrificed HF/HCD-fed mice 2 h after refeeding. While 4-h fasting in HF/HCD-fed Atgl iTg mice led to reduced TG and slightly increased plasma cholesterol concentrations (Table 2), 2-h refeeding resulted in decreased plasma TC and CE levels, which was attributable to less HDL cholesterol (Table 2, Fig. 7A). TG and TC concentrations in the jejunum of Atgl iTg mice were reduced by 31% and 42%, respectively (Fig. 7B). Compared to the 4-h fasted state (Fig. 6B), jejunal TG levels were 4.7-fold higher after 2 h of refeeding. Decreased mRNA expression of jejunal *Cd36, Abca1*, and *ApoA1* in refed Atgl iTg mice (Fig. 7C) was comparable to our results after acute lipid challenge in chow diet-fed mice (Fig. 3F). Unaltered expression of *Hmgcr*, the rate-limiting enzyme in *de novo* cholesterol synthesis, together with comparable *Acat2* expression (Fig. 7C) again suggested that ATGL has no effect on cholesterol synthesis or esterification in the SI upon chronic lipid load.

Cholesterol homeostasis within the SI is a tightly regulated process involving nuclear receptors such as LXR or PPARs, the latter being activated by FFAs that may originate from ATGL-mediated hydrolysis. Therefore, we next investigated the expression of LXR, the three isoforms of PPAR (α, β/δ, and γ), and the farnesoid X receptor (FXR). In the jejunum of Atgl iTg mice, we observed a slight increase in *Ppara* expression, indicating activation by ATGL-derived FFAs (Fig. 7D). In addition, Atgl iTg mice showed a decrease in mRNA expression of Lxr and *Pparβ/δ* by 21% and 33%, respectively, whereas Fxr expression was unchanged. Subsequently, the expression of LXR (*Abca1*, *Abcg1*, *Abcg5*) and PPARδ (*Plin2*, *Fabp1*, *Fabp2*, *Angptl4*, *Pgc1α*) target genes was also reduced (Fig. 7C, E).

### 4.7 ATGL overexpression enhances LXR-mediated HDL secretion

**Fig. 8:**
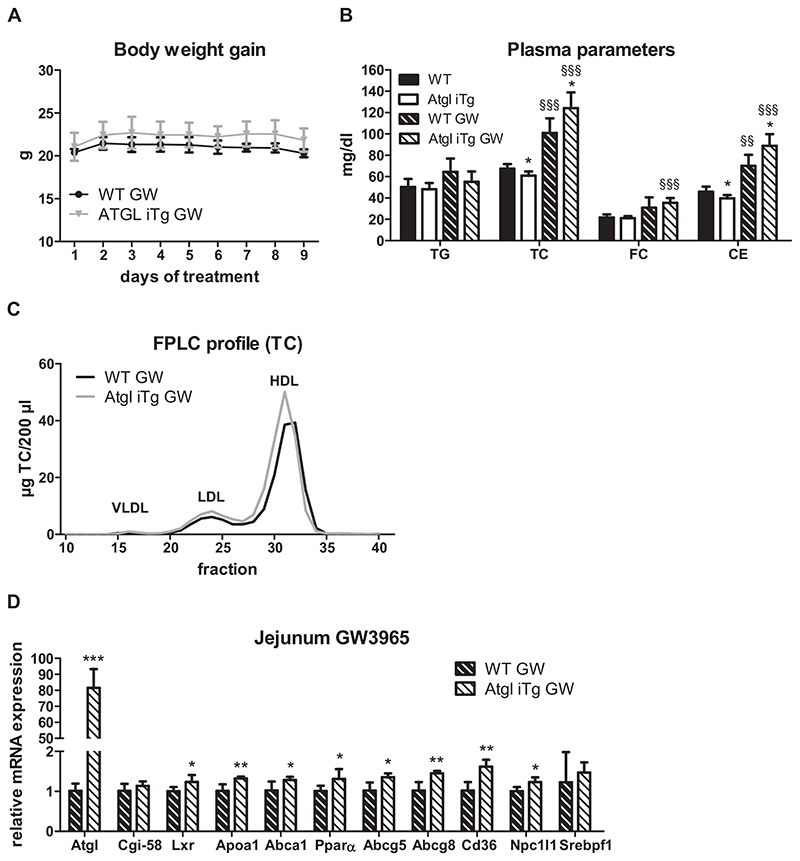
LXR agonist treatment increases plasma HDL in Atgl iTg mice. (A) Chow diet-fed mice were orally treated with the LXR agonist GW3965 (GW) in corn oil containing 1% cholesterol for 9 subsequent days and body weight was monitored daily. Blood was collected from 4-h fasted mice and (B) plasma lipid parameters before and after GW3965 treatment and (C) lipoprotein profiles after 9 days of GW3965 treatment were determined. (D) Jejunal mRNA expression of GW3965-treated WT and Atgl iTg mice normalized to *β-actin* as housekeeping gene. Data represent mean values of 27 week-old female mice (*n* = 4–6) ± SD. * *p* < 0.05; ** *p* ≤ 0.01; *** *p* ≤ 0.001. §§ p ≤ 0.01; §§§ p ≤ 0.001 between pre- and post-treatment within the genotypes.

Finally, we investigated whether ATGL affects LXR activation. For this purpose, we gavaged chow-diet fed mice daily with corn oil containing 1% cholesterol, mimicking HF/HCD feeding, with or without the addition of the LXR agonist GW3965. Oral administration of this agonist was shown to primarily target intestinal LXR without affecting hepatic LXR [25]. While vehicle-treated mice had similar body weight throughout the 9-day treatment (Supplemental Fig. S4A), GW3965-treated Atgl iTg mice tended to gain slightly more weight compared to their WT littermates (Fig. 8A). Baseline plasma cholesterol concentrations were moderately reduced in Atgl iTg mice (Fig. 8B, Supplemental Fig. S4A), consistent with the results in chow diet-fed mice after 4 h of fasting (Table 1, Fig. 2E). As expected, LXR agonist treatment resulted in a significant rise in plasma cholesterol levels in both WT and Atgl iTg mice with no effect on circulating TG levels (Fig. 8B). However, slightly elevated plasma cholesterol levels after 9 days of vehicle-treatment indicated that this rise in cholesterol is partly diet-dependent (Supplemental Fig. S4B). Of note, Atgl iTg mice displayed significantly reduced circulating cholesterol after 9 days of vehicle treatment (Supplemental Fig. S4B, C), which was comparable to results obtained after a single oral lipid bolus (Fig. 3G, Table 1).

Although treatment with GW3965 increased plasma cholesterol concentrations in both genotypes, TC levels were 23% higher in Atgl iTg mice, primarily due to an increase in HDL (Fig. 8C), consistent with increased expression of *Npc1l1, Cd36, Apoa1*, and *Abcal* (Fig. 8D). These results indicated an accelerated dietary cholesterol uptake as observed in HF/HCD-fed Atgl iTg mice (Fig. 5). The slight increase of solely *Ppara* and *Abcg5/g8* in vehicle-treated Atgl iTg mice further suggested an LXR-independent adaptation of these mice to oil-feeding (Supplemental Fig. S4D), which may be responsible for the diminished cholesterol secretion into the plasma.

Compared to vehicle controls, GW3965 treatment drastically induced gene expression of *Abca1, Pparα, Abcg5/g8, Cd36, and Srebf1* in both WT and Atgl iTg mice, without affecting the expression levels of *Atgl, Cgi-58, ApoA1*, and *Npc1l1* (Supplemental Fig. S4E, F), indicating that the latter are not intestinal LXR targets.

In summary, these data indicate that ATGL overexpression impacts LXR signaling in the SI by a yet unknown mechanism, consequently affecting systemic HDL concentrations.

## 5 Discussion

The underlying mechanisms of intestinal LD mobilization and the role of ATGL in this process are insufficiently understood. Using mice overexpressing ATGL exclusively in the SI, we demonstrated that FFAs released by ATGL-mediated hydrolysis are used either for signaling pathways or for energy production, which strongly depends on the nutritional status. Postprandially, FFAs released by ATGL-mediated LD degradation are predominantly shuttled to FAO to meet the increased energy demand during nutrient absorption. Under fasting conditions, FFAs released by intestinal ATGL primarily activate PPARα signaling pathways that increase dietary cholesterol uptake and promote intestinal HDL secretion.

Despite drastically increased mRNA and protein expression, the enzymatic activity of ATGL was only slightly elevated in the jejunum of Atgl iTg mice fed standard chow diet. It has been previously demonstrated that the level of ATGL expression does not necessarily correlate with intracellular enzymatic activity, as isoproterenol- and TNFα-treated adipocytes exhibited unaffected lipase activity levels despite markedly reduced *Atgl* mRNA expression [31]. Thus, post-translational modifications and interactions with other proteins must be considered in our transgenic mice. Due to the limited availability of its coactivator CGI-58, neutral lipolysis may be comparable to that of WT mice. However, the enzymatic activity of ATGL was highly increased in Atgl iTg mice following HF/HCD feeding, which was supported by a concomitant induction of *Cgi-58* mRNA expression and a low abundance of the endogenous inhibitor *G0s2*. This finding is consistent with intestinal ATGL expression being triggered by refeeding or chronic lipid exposure [27,28],

In line with its major role in TG catabolism, ATGL overexpression in enterocytes ameliorated diet-induced intestinal steatosis. Since we observed quite low basal ATGL mRNA and protein expression in the SI, we challenged mice with lipids either acutely (gavage) or chronically (HF/HCD feeding). After an acute lipid load, Atgl iTg mice showed drastically reduced jejunal TG concentrations. Together with decreased expression of *Plin2* and elevated circulating free glycerol, this suggested induced ATGL-mediated lipid hydrolysis in transgenic mice. Consistently, long-term HF/HCD feeding also resulted in massively decreased TG accumulation in the SI of Atgl iTg mice, which was probably facilitated by the reduced abundance of PLIN2 and PLIN3. However, it is also possible that increased LD degradation resulted in diminished PLIN expression. The reduced TG concentrations in the bloodstream may be due to either an increased reuptake of lipids from the circulation or higher utilization of cellular lipids as reflected by increased FAO, independent of dietary lipid intake or CM synthesis. In this context, we have previously demonstrated that intestinal ATGL is predominantly involved in the hydrolysis of a basolaterally derived lipid pool destined for energy production or phospholipid synthesis rather than CM secretion [18]. Therefore, ApoB-dependent TG secretion remained unaffected in chow diet-fed Atgl iTg mice, in agreement with previous observations in intestine- or liver-specific Atgl-deficient mouse models [17,32].

In addition to the effects on intestinal TG catabolism, we also observed modulations of cholesterol homeostasis in the SI of Atgl iTg mice, in line with previous findings in Atgl-deficient enterocytes [17]. Consistent with intestine-specific KO mice exhibiting impaired dietary cholesterol uptake and decreased expression levels of *Cd36* and *Abca1* [17], HF/HCD-fed Atgl iTg mice had accelerated cholesterol uptake from the diet, as shown by decreased radioactivity in feces and elevated tracer concentrations in the circulation. This was presumably caused by increased FFA release from ATGL-mediated LD degradation, which activated *PPARα* and its target genes *Cd36, Npc1l1, Apoa1*, and *Abca1*. Despite increased dietary cholesterol secretion, the unchanged TG incorporation into CMs, along with comparable intestinal *Mttp* expression, indicates no direct effect of ATGL overexpression on ApoB-dependent lipoprotein secretion, but rather cholesterol release *via* HDL. Interestingly, HF/HCD-fed Atgl iTg mice also displayed increased secretion of cholesterol in VLDL-sized particles in the fasted state. Since enterocytes contribute only about 11% to fasted plasma lipid levels [33], elevated VLDL-cholesterol concentrations suggest an effect of intestinal ATGL on hepatic VLDL metabolism. This could be explained in part by increased CM-cholesterol secretion from the SI of Atgl iTg mice, which in turn allows more dietary cholesterol to enter the liver, as shown by the accumulation of radioactive cholesterol in hepatic tissue. Since deletion of the major cholesterol importer NPC1L1 or its pharmacological inhibition with ezetimibe also lowers VLDL-cholesterol levels [34], increased *Npc1l1* expression in HF/HCD-fed Atgl iTg mice could contribute to this phenomenon.

Despite accelerated cholesterol absorption in HF/HCD-fed Atgl iTg mice, modulation of intestinal LXR and PPARβ/δ in refed Atgl iTg mice manifested in diminished circulating cholesterol levels, mainly attributable to reduced HDL cholesterol. We observed an increased deposition of cholesterol after an acute lipid load even in chow diet-fed Atgl iTg mice, as also demonstrated by accumulation of radiolabeled or fluorescently labeled cholesterol. Comparable incorporation of acetic acid into cholesterol excluded the possibility of altered *de novo* cholesterol synthesis in these mice. However, downregulation of genes involved in ApoB-independent HDL secretion in the SI (*Apoa1, Abca1*) may be responsible for this effect. Of note, the reduction in circulating cholesterol was even more pronounced in HF/HCD-fed Atgl iTg mice in the refed state. Decreased HDL cholesterol was in accordance with the modulation of LXR and PPARβ/δ target genes, both of which play important roles in intestinal HDL synthesis and secretion [25,35]. Since activation of LXR leads to an increase in HDL cholesterol through upregulation of intestinal *Abca1* [25], the decrease in its expression may be responsible for the reduced plasma HDL cholesterol in Atgl iTg mice. PPAR response elements (PPRE) are present in the promotor region of the LXR gene, leading to an activation of PPARα and, further, LXR in macrophages and hepatocytes (reviewed by [36]). In the SI, LXR reciprocally regulates the expression of PPARα but no other PPARs [37]. Therefore, decreased mRNA expression of intestinal *Pparβ/δ* is independent of LXR but may also contribute to the decrease in circulating HDL of Atgl iTg mice, in line with elevated HDL cholesterol and impaired cholesterol absorption by PPARβ/δ agonist treatment [35], most likely through upregulation of *Abca1* and downregulation of *Npc1l1*, respectively. Moreover, the regulation of ATGL and PPARs is highly interdependent, with PPARγ inducing ATGL expression, thereby promoting the release of FFAs to activate PPARα and/or PPARβ/δ (reviewed by [38]) and consequently FAO in Atgl iTg mice. Although speculative, pronounced activation of PPARα may counteract the activation of PPARβ/δ and its target genes due to limited availability of endogenous ligands. Nevertheless, how ATGL affects LXR signaling remains elusive. Although some studies have demonstrated increased ATGL expression upon pharmacological LXR activation [39,40], LXR has no direct effect on ATGL expression [41].

Treatment with the LXR agonist GW3965 increased not only LXR target genes but also intestinal *Pparα*, which was in line with previous findings [37], however, unaffected expression of *Npc1l1* and *ApoA1* after GW3965 treatment indicated that these genes are no targets of intestinal LXR. This result was contradictory to previously studies showing that *in vivo* treatment with LXR agonists upregulates *Abcg5/g8* in the SI [42] and decreases *Npc1l1* expression, resulting in reduced intestinal cholesterol absorption [43]. Although systemic cholesterol concentrations were increased in both genotypes upon LXR activation, over-expression of ATGL potentiated this effect, resulting in increased dietary cholesterol absorption. GW3965-treated Atgl iTg mice displayed elevated circulating TC and CE levels mainly due to higher HDL-cholesterol concentrations, accompanied by increased intestinal expression of genes involved in dietary cholesterol uptake (*Cd36*, *Npc1l1*) and intestinal HDL secretion (*Abca1, Apoa1*). Although one might have expected diminished LXR activation resulting in impaired HDL cholesterol secretion, as observed in refed Atgl iTg mice, this finding was in line with accelerated cholesterol absorption in HF/HCD-fed mice. However, it is questionable to compare intragastric lipid load with chronic HF/HCD feeding and fasted or refed conditions. Of note, and consistent with a single oral lipid bolus, vehicle-treated Atgl iTg mice displayed reduced abundance of HDL cholesterol levels after 9 days. Whereas the reduction in circulating cholesterol concentrations after a single oil bolus was mainly attributable to diminished *ApoA1* and *Abca1* mRNA expression, chronic intragastric oil feeding led to adaptations in cholesterol excretion by upregulating *Abcg5/g8*. This slight increase in *Abcg5/g8* expression in vehicle-treated Atgl iTg mice further suggested that overexpression of ATGL impacts dietary cholesterol excretion *via* an LXR-independent mechanism that likely involves PPARα.

Since ATGL is regulated differently in the SI compared to other tissues, the underlying mechanism of its modulations under fed and fasting conditions and its involvement in LXR signaling require further investigation. One might speculate that the differences between fasted HF/HCD-fed mice and mice sacrificed in the refed state after acute or chronic lipid load could be an altered lymphatic function, because postprandial lipids have been shown to diminish lymphatic contractile function [44]. However, a recent study demonstrated that intestinal HDL in mice does not enter the lymphatics but the portal vein [11], implying that changes in the lymphatic function should not affect HDL cholesterol levels in Atgl iTg mice. Furthermore, it is important to consider that intestinal ATGL expression is strongly influenced by the nutritional status: Intestinal ATGL expression is induced by chronic (diet-induced obesity) and acute (after food intake) lipid stress after 6 h of fasting but reduced by ~80% 2 h after oil gavage of diet-induced obese mice [28]. In this context, one might argue that especially during HF/HCD feeding, 4-h fasting or 2-h of refeeding could completely reverse the phenotype of Atgl iTg mice.

We conclude that FFAs released by ATGL-mediated hydrolysis in the postprandial state are shuttled toward FAO to generate energy for the highly energy-consuming nutrient absorption process, resulting in limited ligand availability for PPARβ/δ activation and impairing intestinal HDL metabolism. In contrast, FFAs released by intestinal ATGL during fasting primarily activate PPARα signaling pathways that increase dietary cholesterol uptake and promote intestinal HDL secretion, with additional involvement of LXR. However, the interplay between ATGL and LXR in enterocytes needs further investigation.

## Supplementary Material

Table S1, Figures S1 - S4

## Figures and Tables

**Table 1:** Body weight and plasma lipid levels of chow diet-fed WT and Atgl iTg mice.

	Chow (16 h fasted)		Chow (4 h fasted)		Chow (ad libitum fed)	
	WT	iTg	WT	iTg	WT	iTg
BW (g)	n.d.	n.d.	17.8 ±0.52	19.2 ±1.65	16.6 ±0.31	16.2 ±0.57
TG (mg/dl)	55.5 ±8.19	59.3 ±10.18	65.9 ±6.28	67.3 ±11.1	43.4 ±2.60	46.4 ±3.93
TC (mg/dl)	56.6 ±5.00	50.2 ±2.41 *	68.3 ±5.34	57.5 ±6.30 *	63.2 ±6.46	52.1 ±1.72 *
FC (mg/dl)	21.6 ±2.71	19.8 ±0.95	19.6 ±4.26	19.4 ±1.75	18.3 ±3.43	15.8 ±1.18
CE (mg/dl)	35.1 ±3.73	30.3 ±2.11 *	48.8 ±4.70	38.2 ±7.52 *	44.9 ±3.57	36.3 ±2.14 **
FFA (mmol/l)	n.d.	n.d.	0.48 ±0.18	0.50 ±0.08	n.d.	n.d.
FG (mg/dl)	n.d.	n.d.	4.05 ±0.40	4.18 ±0.58	n.d.	n.d.
n	7	6	6	5	5	5
	Chow (refed)		Chow (2 h p.g.)		Chow (4 h p.g.)	
	WT	iTg	WT	iTg	WT	iTg
BW (g)	16.4 ±0.10	16.6 ±0.64	n.d.	n.d.	n.d.	n.d.
TG (mg/dl)	48.7 ±7.33	44.7 ±2.30	54.5 ±8.64	47.8 ±7.43	53.0 ±3.51	68.8 ±9.05 *
TC (mg/dl)	92.6 ±7.74	74.0 ±2.42 *	59.7 ±5.15	51.0 ±6.13 *	66.8 ±3.44	62.2 ±3.59
FC (mg/dl)	27.8 ±2.72	21.3 ±2.46	24.1 ±0.75	23.3 ±3.26	24.2 ±1.36	27.5 ±4.40
CE (mg/dl)	64.8 ±9.52	52.7 ±4.38	34.5 ±4.48	27.8 ±3.03 *	42.6 ±2.46	34.8 ±3.03 **
FFA (mmol/l)	2.25 ±0.19	2.43 ±0.24	n.d.	n.d.	n.d.	n.d.
FG (mg/dl)	0.19 ±0.01	0.24 ±0.00 ***	n.d.	n.d.	n.d.	n.d.
n	4	4	7	6	4	4

Body weight and lipid parameters of female age-matched chow diet-fed WT and Atgl iTg mice in fed and fasted conditions. Data were analyzed by unpaired Student’s t-test. Data represent mean (n = 4-7) ± SD. BW, body weight; TG, triglyceride; TC, total cholesterol; FC, free cholesterol; CE, cholesteryl ester calculated by subtracting FC from TC; FFA, free fatty acid; FG, free glycerol. n.d., not determined.

* p < 0.05.

** p ≤ 0.01.

*** p ≤ 0.001.

**Table 2:** Body weight and plasma lipid levels of HF/HCD-fed WT and Atgl iTg mice.

	5w HF/HCD (4 h fasted)		4w HF/HCD (ad libitum fed)		8w HF/HCD (refed)	
	WT	iTg	WT	iTg	WT	iTg
BW (g)	20.1 ±1.63	21.2 ±1.82	18.0 ±0.36	20.0 ±3.05	21.1 ±0.39	21.5 ±1.05
TG (mg/dl)	41.1 ±5.73	40.7 ±5.89	71.0 ±18.3	56.5 ±13.8	67.0 ±8.87	72.6 ±9.62
TC (mg/dl)	80.8 ±13.4	95.1 ±18.5	131 ±7.52	112 ±9.50 **	111 ±12.2	94.6 ±2.69 *
FC (mg/dl)	35.1 ±4.94	31.5 ±4.46	32.5 ±1.48	28.6 ±1.56	34.4 ±5.03	34.6 ±1.58
CE (mg/dl)	45.7 ±13.3	63.6 ±15.8 *	98.6 ±6.66	81.4 ±13.1 *	76.2 ±11.5	60.0 ±4.10 *
FFA (mmol/l)	0.29 ±0.08	0.29 ±0.08	n.d.	n.d.	2.84 ±0.17	3.16 ±0.22
FG (mg/dl)	3.99 ±0.64	3.57 ±0.51	n.d.	n.d.	0.51 ±0.03	0.52 ±0.04
n	10	10	5	5	5	5

Body weight and lipid parameters of female age-matched WT and Atgl iTg mice in fed or fasted conditions after HF/HC diet feeding for 4-8 weeks. Data were analyzed by unpaired Student’s t-test. Data represent mean (*n* = 5-10) ± SD. BW, body weight; TG, triglyceride; TC, total cholesterol; FC, free cholesterol; CE, cholesteryl ester calculated by subtracting FC from TC; FFA, free fatty acid; FG, free glycerol.

n.d., not determined.

* p < 0.05.

** p ≤ 0.01.

## Abbreviations

ATGL: adipose triglyceride lipase
HDL: high-density lipoprotein
LXR: liver X receptor
PPAR: peroxisome proliferator-activated receptor.
