# Supplementary material for: Enterocyte-specific ATGL overexpression affects intestinal and systemic cholesterol homeostasis
Source: Biochim Biophys Acta Mol Cell Biol Lipids. Author manuscript; Available in PMC 2022 Nov 30. (PMC7613867; doi:10.1016/j.bbalip.2022.159121)
Supplement: Table S1, Figures S1 - S4 [file EMS157444-supplement-Table_S1__Figures_S1___S4.pdf]

Supplementary material

Enterocyte-specific ATGL overexpression affects intestinal and systemic cholesterol homeostasis

Melanie Korbelius, Nemanja Vujić, Katharina B. Kuentzel, Sascha Obrowsky, Silvia Rainer, Guenter Haemmerle, Thomas Rülicke, and Dagmar Kratky

Table S1: Primer sequences

Gene	Forward primer	Reverse primer
Abca1	CTCTTCATGACTCTAGCCTGGA	ACACAGACAGGAAGACGAACAC
Abcg1	CTTTCCTACTCTGTACCCGAGG	CGGGGCATTCCATTGATAAGG
Abcg5	AGAGGGCCTCACATCAACAGA	CTGACGCTGTAGGACACATGC
Abcg8	CTGTGGAATGGGACTGTACTTC	GTTGGACTGACCACTGTAGGT
Acat2	GATGGTCTGACAGATGCCTT	AGCACTGGCACAATCTCTT
Angptl4	GCATCCTGGGACGAGATGAAC	CATGGCCGAGCTGTAGCAG
Atgl	GCCACTCACATCTACGGAGC	GACAGCCACGGATGGTGTTC
Apoa1	AGCTGAACCTGAATCTCCTG	CACTTCCTCTAGGTCCTTGT
β-actin	CGGTTCCGATGCCCTGAGGCTCTT	CGTCACACTTCATGATGGAATTGA
Cd36	GCAGGTCTATCTACGCTGTG	GGTTGTCTGGATTCTGGAGG
Cgi-58	GGTTAAGTCTAGTGCAGC	AAGCTGTCTCACCACTTG
CyclophilinA	CCATCCAGCCATTCAGTCTT	TTCCAGGATTCATGTGCCAG
Emr1	CTTTGGCTATGGGCTTCCAGTC	GCAAGGAGGACAGAGTTTATCGTG
Fabp1	CTTCTCCGGCAAGTACCAAT	GAACTCATTGCGGACCACTT
Fabp2	ACTAATCCAGACCTACACATATGAAGGA	GCTCCAGGCTCTGAGAAGTTGA
Fxr	GCTTGATGTGCTACAAAAGCTG	CGTGGTGATGGTTGAATGTCC
G0s2	GTGAAGCTATACGTGCTGGG	CCGTCTCAACTAGGCCGAG
Hmgcs	CGGATCGTGAAGACATCAACTC	CGCCCAATGCAATCATAGGAA
Hmgcr	TGTTCACCGGCAACAACAAGA	CCGCGTTATCGTCAGGATGA
Hsl	GCTGGTGACACTCGCAGAAG	TGGCTGGTGTCTCTGTGTCC
Ldlr	CTCCTGCATTCACGGTAGCC	CCCACTGTGACACTTGAACTTG
Lrp1	CCACTATGGATGCCCCTAAAAC	GCAATCTCTTTCACCGTCACA
Lxr	CTCAATGCCTGATGTTTCTCCT	TCCAACCCTATCCCTAAAGCAA
Mttp	GTCAACAGAGAGGCGAGAAG	CTAGCCAAGCCTCTCTTGAG
Npc1l1	GCAAGGTGATCAGGAGGTTGA	ATCCTCATCCTGGGCTTTGC
Pgc1α	CCCTGCCATTGTTAAGACC	TGCTGCTGTTCCTGTTTTC
Plin2	GACCTTGTGTCCTCCGCTTAT	CAACCGCAATTTGTGGCTC
Plin3	ATGTCTAGCAATGGTACAGATGC	CGTGGAACTGATAAGAGGCAGG
Pparα	TTCACAAGTGCCTGTCTGTC	GGCCTTGACCTTGTTCATGT
Pparβ/δ	GCAGCCTCAACATGGAATGTC	GAGCTTCATGCGGATTGTCC
Pparγ	GGAAGACCACTCGCATTCCTT	GTAATCAGCAACCATTGGGTCA
Scarb1	TTTGGAGTGGTAGTAAAAAGGGC	TGACATCAGGGACTCAGAGTAG
Srebf1	CACTCAGCAGCCACCATCTAGCCT	GCTGATGCCTGCAGTCTTCACG
Vldl-R	GAGTCTGACTTCGTGTGCAAA	GAACCGTCTTCGCAATCAGGA

Figure S1

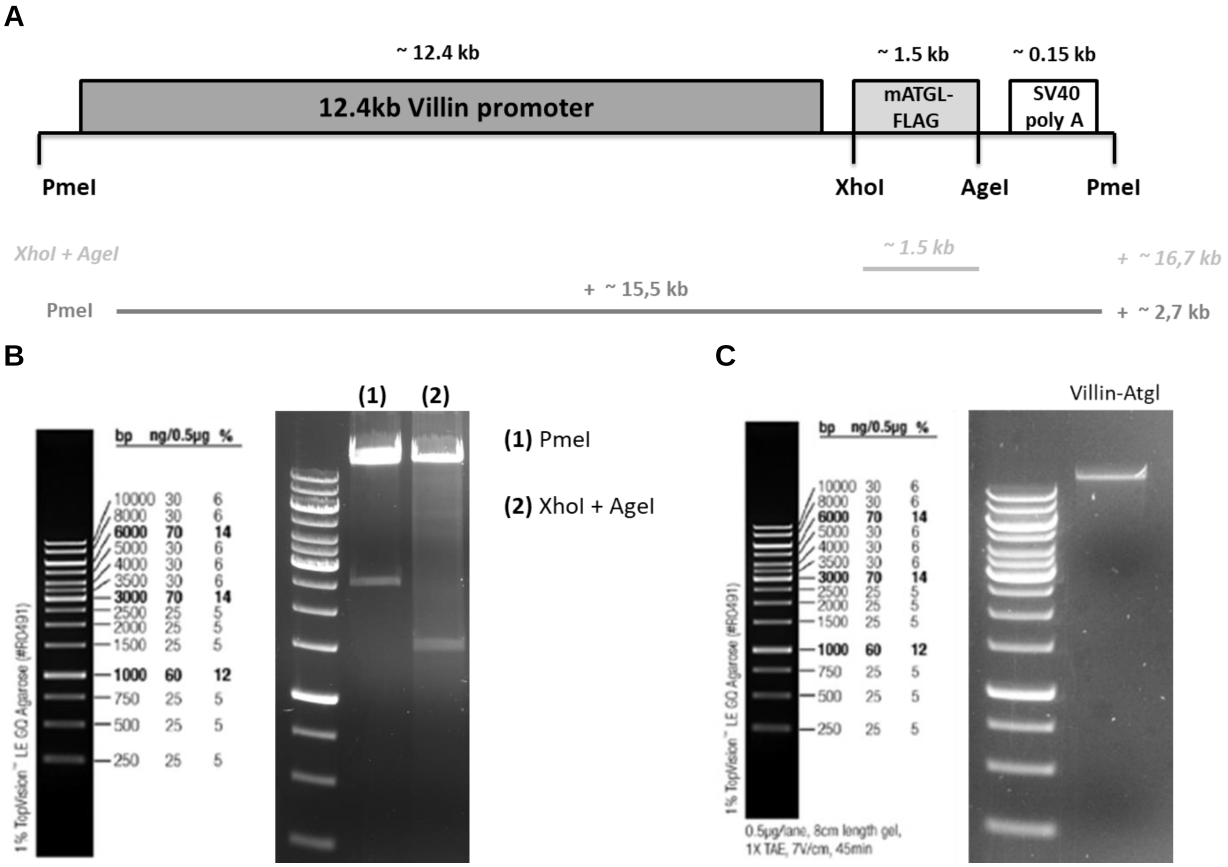

Figure S1: Schematic overview of the Atgl transgene used to generate Atgl iTg mice. (A) C-terminal FLAG-tagged mouse *Atgl* cDNA was cloned downstream of the intestine-specific villin promotor sequence and upstream of the SV40 poly(A) signal with a size of 15.5 kb. (B) Control digest with PmeI yielding the functional construct (15.5 kb) and the vector backbone (2.7 kb). Digestion with XhoI and AgeI resulted in excision of the inserted ATGL construct (1.5 kb) and the residual vector (16.7 kb). (C) Purification of the linearized construct with a final size of 15.5 kb.

Figure S2

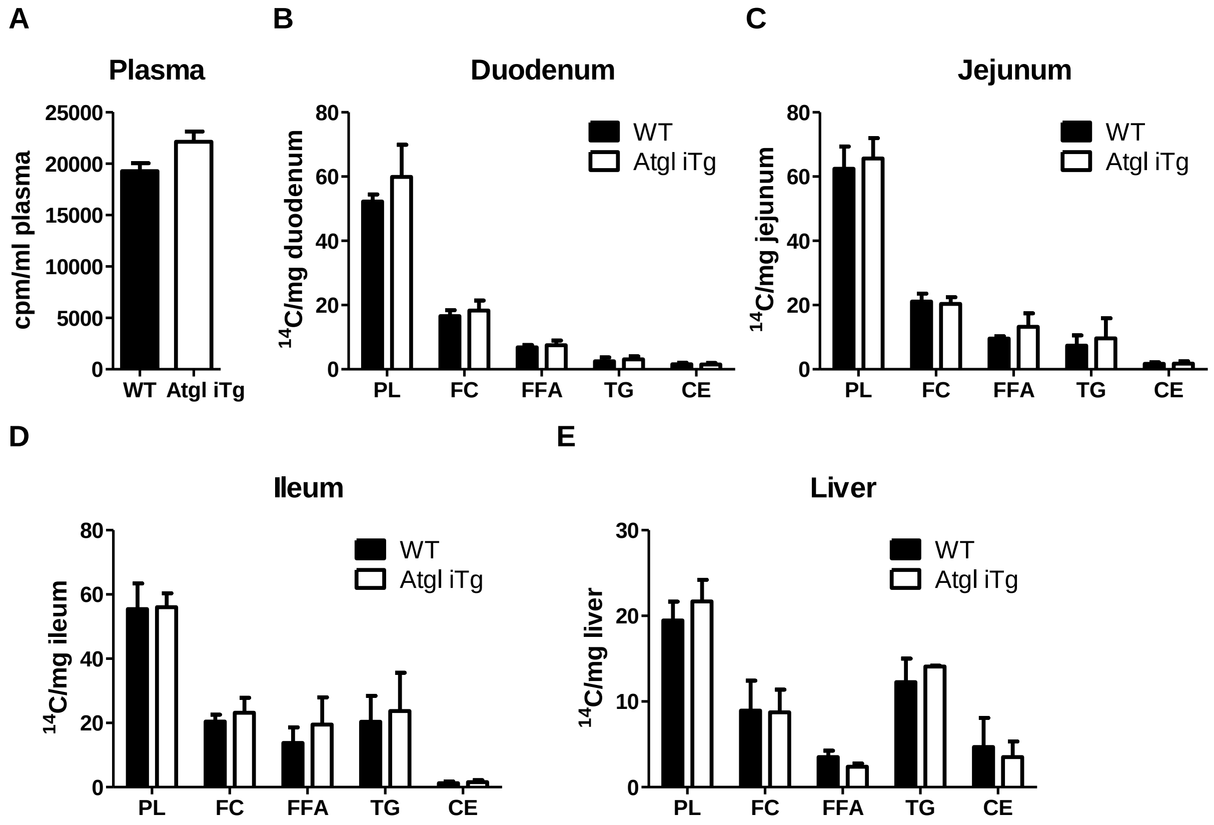

Figure S2: Unchanged *de novo* cholesterol synthesis in Atgl iTg mice. Mice were fasted for 4 h prior to intraperitoneal injection of [^{14}C]-acetate in 200 µl PBS and sacrificed 1 h after injection. (A) Total radioactivity in plasma and (B-E) separation of intestinal and hepatic tissue samples by TLC. PL, phospholipids; FC, free cholesterol; FFA, free fatty acids; TG, triglycerides; CE, cholesteryl esters. Data represent mean values of 21 week-old female mice (n=3-4) + SD.

Figure S3

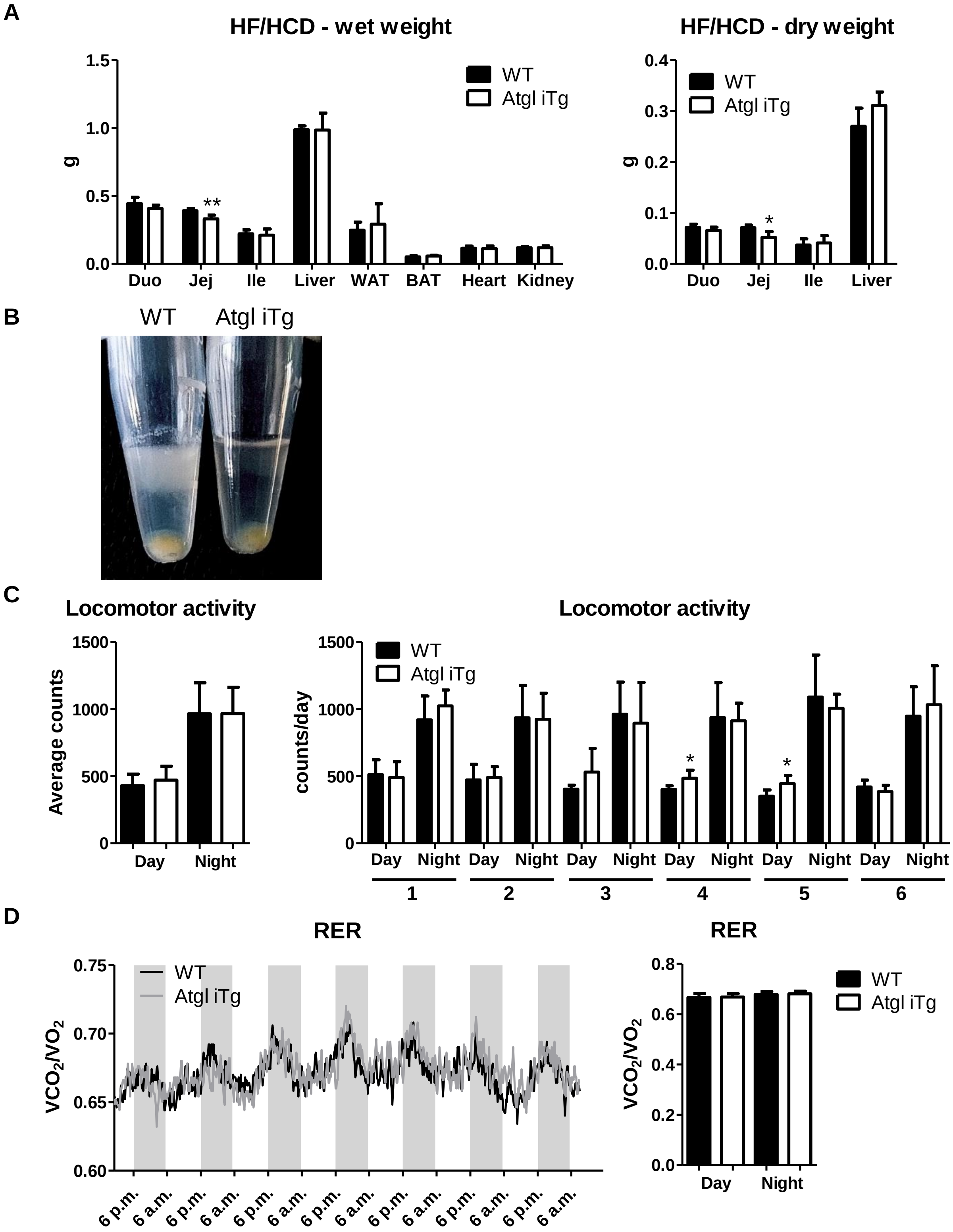

Figure S3: Decreased jejunal weight in Atgl iTg mice. (A) Wet and dry weight of intestinal and hepatic tissues of WT and Atgl iTg mice fed HF/HCD for 12 weeks. (B) Jejunal tissue lysates after centrifugation for 30 min at 14,000 rpm. After 10 weeks of HF/HCD feeding, mice were housed in metabolic cages to monitor (C) locomotor

activity and (D) respiratory exchange rate (RER). Data represent mean of 16-18 week-old female mice (n=4-5) + SD. * $p < 0.05$; ** $p \leq 0.01$.

Figure S4

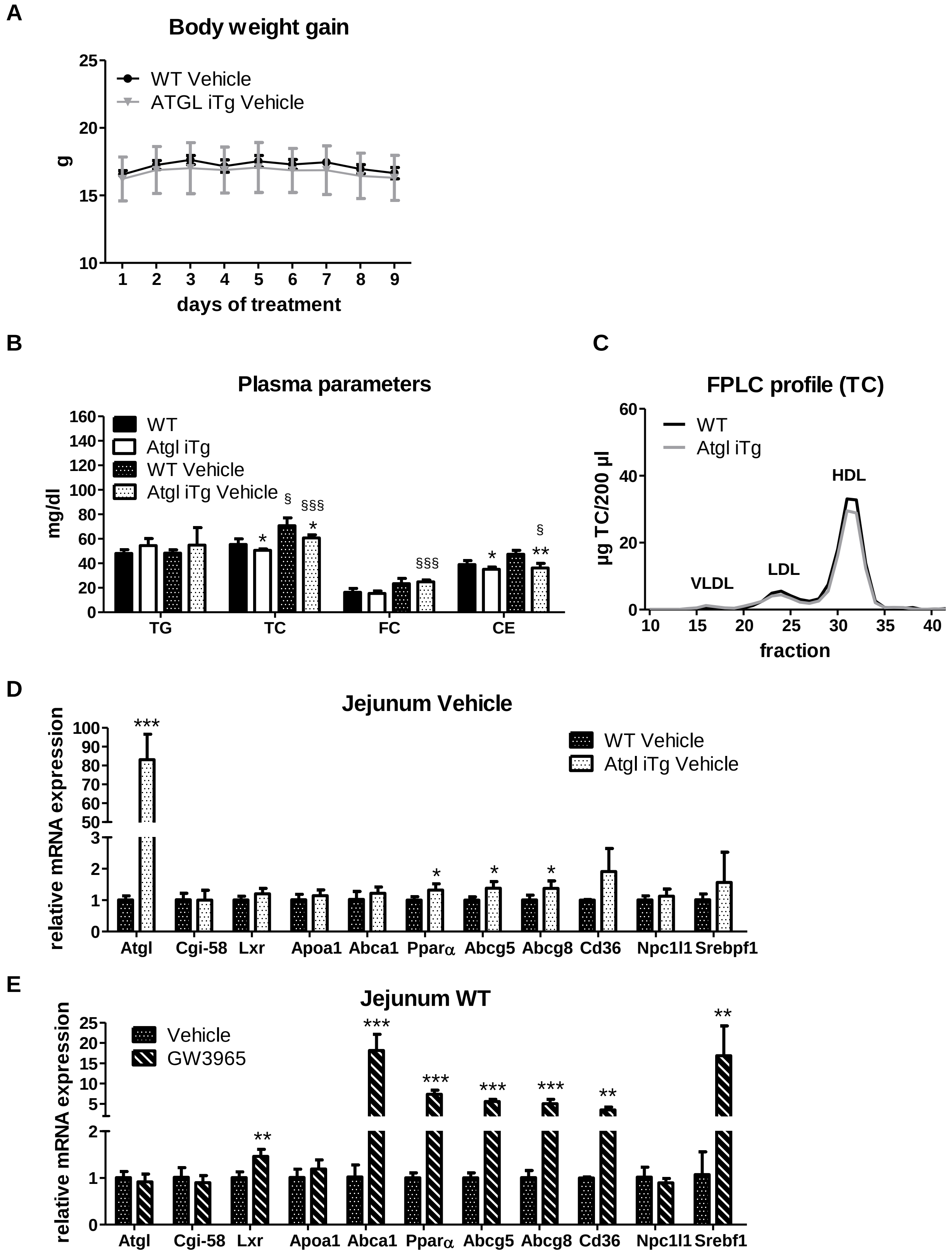

A
Body weight gain
WT Vehicle
ATGL iTg Vehicle
g
days of treatment
B
Plasma parameters
WT
Atgl iTg
WT Vehicle
Atgl iTg Vehicle
mg/dl
TG
TC
FC
CE
C
FPLC profile (TC)
WT
Atgl iTg
µg TC/200 µl
VLDL
LDL
HDL
fraction
D
Jejunum Vehicle
WT Vehicle
Atgl iTg Vehicle
relative mRNA expression
Atgl Cgi-58 Lxr Apoa1 Abca1 Pparα Abcg5 Abcg8 Cd36 Npc1l1 Srebpf1
E
Jejunum WT
Vehicle
GW3965
relative mRNA expression
Atgl Cgi-58 Lxr Apoa1 Abca1 Pparα Abcg5 Abcg8 Cd36 Npc1l1 Srebf1

F

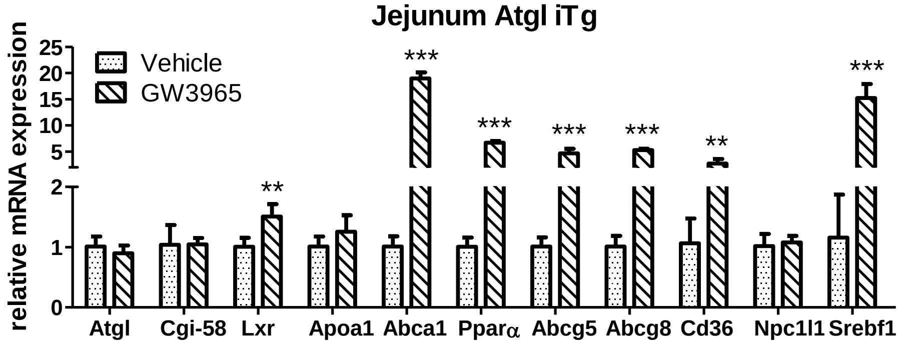

Figure S4: GW3965 treatment induces jejunal *Abca1* expression. Chow diet-fed mice were orally treated with corn oil containing 1% cholesterol in the absence (vehicle) or presence of the LXR agonist GW3965 for 9 subsequent days. (A) Body weight gain during vehicle treatment, (B) plasma lipid parameters before and after vehicle treatment, and (C) lipoprotein profile of WT and Atgl iTg mice after vehicle treatment. (D) Jejunal mRNA expression profile in vehicle-treated WT and Atgl iTg mice normalized to *β-actin* as housekeeping gene. (E, F) Effect of GW3965 treatment on jejunal gene expression in (E) WT and (F) Atgl iTg mice normalized to *β-actin* as housekeeping gene. Data represent mean values of 11 (vehicle) and 27 (GW3965) week-old female mice (n=4-6) ± SD. * $p < 0.05$; ** $p \le 0.01$; *** $p \le 0.001$. § $p < 0.05$; §§§ $p \le 0.001$ between pre- and post-treatment within the genotypes.
